# Targeted Ablation of *Crb1* and *Crb2* in Retinal Progenitor Cells Mimics Leber Congenital Amaurosis

**DOI:** 10.1371/journal.pgen.1003976

**Published:** 2013-12-05

**Authors:** Lucie P. Pellissier, Celso Henrique Alves, Peter M. Quinn, Rogier M. Vos, Naoyuki Tanimoto, Ditte M. S. Lundvig, Jacobus J. Dudok, Berend Hooibrink, Fabrice Richard, Susanne C. Beck, Gesine Huber, Vithiyanjali Sothilingam, Marina Garcia Garrido, André Le Bivic, Mathias W. Seeliger, Jan Wijnholds

**Affiliations:** 1 Department of Neuromedical Genetics, The Netherlands Institute for Neuroscience, Royal Netherlands Academy of Arts and Sciences (KNAW), Amsterdam, The Netherlands; 2 Division of Ocular Neurodegeneration, Institute for Ophthalmic Research, Centre for Ophthalmology, Eberhard Karls University of Tübingen, Tübingen, Germany; 3 Department of Cell Biology and Histology, Amsterdam Medisch Centrum, Amsterdam, The Netherlands; 4 Aix-Marseille University, Developmental Biology Institute of Marseille Luminy (IBDML) and CNRS, UMR 6216, Marseille, France; Johns Hopkins University School of Medicine, United States of America

## Abstract

Development in the central nervous system is highly dependent on the regulation of the switch from progenitor cell proliferation to differentiation, but the molecular and cellular events controlling this process remain poorly understood. Here, we report that ablation of *Crb1* and *Crb2* genes results in severe impairment of retinal function, abnormal lamination and thickening of the retina mimicking human Leber congenital amaurosis due to loss of *CRB1* function. We show that the levels of CRB1 and CRB2 proteins are crucial for mouse retinal development, as they restrain the proliferation of retinal progenitor cells. The lack of these apical proteins results in altered cell cycle progression and increased number of mitotic cells leading to an increased number of late-born cell types such as rod photoreceptors, bipolar and Müller glia cells in postmitotic retinas. Loss of CRB1 and CRB2 in the retina results in dysregulation of target genes for the Notch1 and YAP/Hippo signaling pathways and increased levels of P120-catenin. Loss of CRB1 and CRB2 result in altered progenitor cell cycle distribution with a decrease in number of late progenitors in G1 and an increase in S and G2/M phase. These findings suggest that CRB1 and CRB2 suppress late progenitor pool expansion by regulating multiple proliferative signaling pathways.

## Author Summary

Mutations in the human *CRB1* gene lead to one of the most severe forms of retinal dystrophies, called Leber congenital amaurosis. Here, we report that ablation of CRB1 and the second family member CRB2 are crucial for proper retinal development. These mice display severe impairment of retinal function, abnormal lamination and thickening of the retina mimicking human Leber congenital amaurosis due to loss of *CRB1* function. The thickening of the retina is due to increased cell proliferation during late retinal development leading to an increased number of late-born retinal cells. We describe in these *CRB1* Leber congenital amaurosis mouse models the molecular and cellular events involving CRB proteins during the development of the retina.

## Introduction

During vertebrate retina development, one type of glial cell and six types of neurons are formed by the orderly generation of post-mitotic cells from a common pool of retinal progenitor cells [1], [2]. In this temporally fine-tuned process, ganglion cells are generated first, followed by horizontal cells, cone photoreceptors and early born amacrine cells, rod photoreceptors and late born amacrine cells, and finally bipolar cells and Müller glial cells [2]. Retinal progenitor cells are elongated and polarized cells that extend along the apicobasal axis and connect to adjoining cells by adherens junctions via their apical processes. The proliferation of the progenitors is carefully regulated through a combination of intrinsic and extrinsic signals followed by a complete cessation of cell division around 10 days after birth in mice [3]. Many extrinsic soluble or membrane-bound factors directly promote proliferation activity such as Notch, sonic Hedgehog and Wnt signalling pathways [4]. In addition, intrinsic regulatory genes and transcription factors such as *Chx10* regulate the cell cycle machinery [5].

Recent work suggests that cell adhesion and cell polarity complex proteins play a critical role in the maintenance of the proliferation of the progenitor cells [6]. The polarity proteins that form the Crumbs complex reside at the subapical region adjacent to the adherens junctions between retinal progenitor cells in the developing retina or between photoreceptors and Müller glial cells in mature retinas. The *Crumbs* protein was first identified in *Drosophila* as a key developmental regulator of apical-basal polarity [7]. In mammals, the Crumbs homologue family is composed of three genes, *CRB1*, *CRB2* and *CRB3*. CRB proteins have a large extracellular domain (which is lacking in CRB3) composed of epidermal growth factor and laminin-globular domains, a single transmembrane domain, and an intracellular domain containing FERM and PDZ protein-binding motifs [8]. Through this PDZ motif CRB proteins interact with PALS1, which binds to MUPP1 or PATJ, thus forming the Crumbs complex [8]. Recently, it has been shown that the CRB-interacting partner PALS1 has a role in regulating the proliferation of neural progenitors. Deletion of PALS1 in the developing cortex caused premature exit of progenitors from the cell cycle and massive cell death leading to absence of the cortical structures [9].

Studies suggest a common function of CRB proteins and their partners in regulating growth factor signalling pathways, which orchestrate cell proliferation and cell fate decisions. It has been suggested that *Drosophila Crumbs* and human CRB2 inhibit Notch1 cleavage and signalling by binding to the presenilin complex, inhibiting γ-secretase activity [10], [11]. Zebrafish CRB extracellular domains can directly bind to the extracellular domain of Notch1 and inhibit its activation [12]. The Crumbs complex can negatively modulate the mammalian Target of Rapamycin Complex 1 (mTORC1) pathway via the direct interaction between PATJ and the tumour suppressor gene TSC2 and depletion of PALS1 protein results in loss of mTORC1 activity in the murine developing cortex [9], [13]. The Hippo pathway is a key regulator of organ size and tumorigenesis in humans and flies [6], [14]. *Drosophila Crumbs* has been shown to control the Hippo pathway by direct interaction of its FERM domain [15], [16]. Furthermore, PALS1 and PATJ can interact with the effectors of the Hippo pathway Yes-associated Protein (YAP) and transcriptional co-activator with PDZ-binding motif (TAZ) proteins and thus promote their inhibition and retention in the cytoplasm [17].

Mutations in the human *CRB1* gene cause autosomal-recessive progressive retinitis pigmentosa and Leber congenital amaurosis (LCA) [18]. LCA is one of the most severe forms of retinal dystrophy leading to blindness around birth due to defects in the development or maturation of the retina [19]. *CRB1*-LCA retinas are remarkably thick and lack the distinct layers like immature retinas suggesting a developmental defect [20]. The functional roles of CRB proteins during mammalian development remain poorly understood. Both CRB1 and CRB2 are expressed from embryonic day (E)12.5 onwards in the developing murine retina at the subapical region adjacent to adherens junctions in retinal progenitor cells [21]–[23] suggesting a role of the CRB proteins during the development of the retina. *Crb1* knockout, *Crb1^C249W/−^* knockin and the naturally occurring *Crb1^rd8/rd8^* mutant mice show mild retinal disorganization in adulthood, limited to the inferior quadrant [24]–[27]. *Crb2* conditional knockout (cKO) retinas show progressive abnormal lamination of newborn rod photoreceptors and disruption of adherens junctions in postnatal developing retina [22]. Here, we study the effects of loss of CRB1 and CRB2 and their potential overlapping functions during early retinal development. Loss of both CRB1 and CRB2 results in absence of a separate photoreceptor layer, misplaced cell types throughout the retina and loss of retinal function mimicking the phenotype observed in human LCA patients. Our data suggests that the pool of late progenitor cells during retinal development is suppressed by CRB1 and CRB2 through the regulation of mitogenic signaling pathways.

## Results

### Lack of CRB1 and CRB2 severely impairs retinal function in adult mice

We crossed *Crb1* KO mice with conditionally floxed *Crb2* mice [22], [24]. The mice were bred with Chx10*Cre* transgenic mice, which express Cre recombinase fused to GFP throughout the developing retina starting at E11.5 [28]. We showed previously that efficient recombination of the floxed *Crb2* alleles occurred around E12.5 [22]. In this study, double homozygote *Crb1^−/−^Crb2^F/F^*Chx10*Cre^Tg/+^* conditional knockout retinas (*Crb1Crb2* cKO) were compared to littermate *Crb1^−/−^Crb2^F/F^* and *Crb1^−/−^Crb2^F/+^*Chx10*Cre^Tg/+^* retinas. *Crb1^+/−^Crb2^F/F^*Chx10*Cre^Tg/+^* (*Crb1^+/−^Crb2* cKO) retinas were compared to littermate double heterozygote *Crb1^−/+^Crb2^F/+^*Chx10*Cre^Tg/+^* (*Crb1^+/−^Crb2^F/+^* cKO) retinas. We verified the loss of CRB1 and CRB2 proteins in the *Crb1Crb2* cKO at E15.5 and P14 (Figures S4D and S3D).

**Figure 1:**
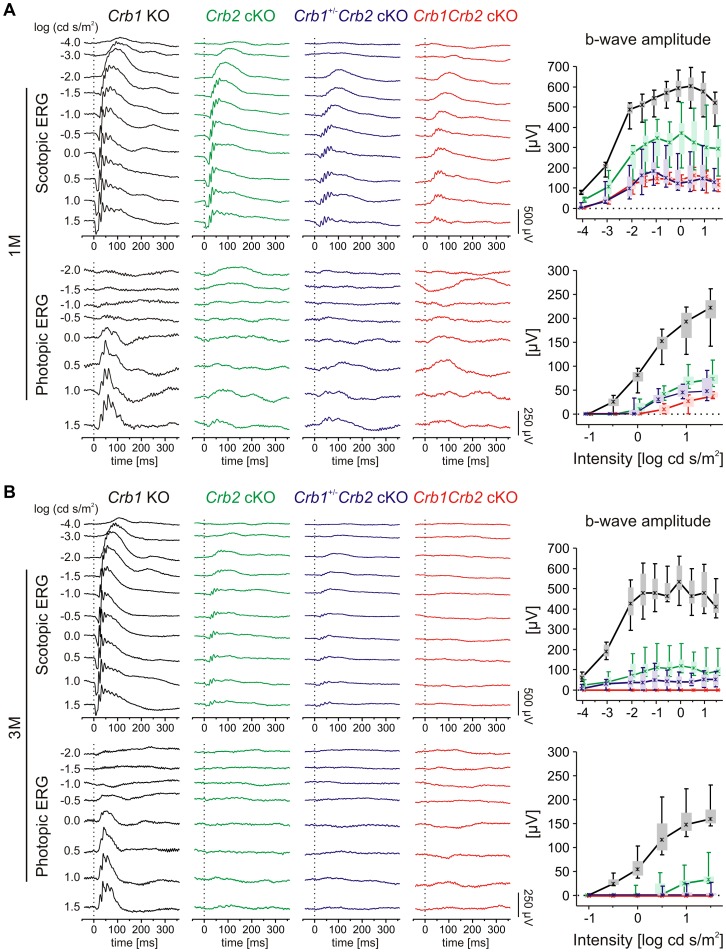
Retinal function in *Crb1Crb2* mutant retinas is severely impaired. Retinal function in *Crb1* KO (black), *Crb2* cKO (green), *Crb1^+/−^Crb2* cKO (purple) and *Crb1Crb2* cKO affected mice (red) based on single-flash electroretinogram data from 1M (A), and 3M (B) old animals. (left) Representative single-flash electroretinogram traces recorded from the indicated genotypes under scotopic (top) and photopic (bottom) conditions. (right) Scotopic (top) and photopic (bottom) b-wave amplitude data plotted as a function of the logarithm of the flash intensity. Boxes indicate the 25% and 75% quantile range, whiskers indicate the 5% and 95% quantiles, and the asterisks indicate the median of the data. In *Crb1^+/−^Crb2* cKO and *Crb1Crb2* cKO mice, the b-wave amplitude was already considerably reduced at 1M under both scotopic and photopic conditions, and declined even at 3M compared to *Crb1* KO and *Crb2* cKO.

*In vivo* functional and structural analysis were performed on 1 to 6 month (M) old *Crb1Crb2* cKO, *Crb1^+/−^Crb2* cKO and control mice, using electroretinography, spectral domain optical coherence tomography and scanning laser ophthalmoscopy. Already at 1M, *Crb1^+/−^Crb2* cKO and *Crb1Crb2* cKO mice showed more pronounced reduction in amplitudes of electroretinogram responses than *Crb2* cKO mice (Figures 1A and S1A). Both scotopic and photopic responses were affected, which indicate alterations of both rod and cone system components. At 3 and 6M (Figures 1B and S1B–C), electroretinogram responses were below detection level, although *Crb1^+/−^Crb2* cKO responses were more variable (Figures 1B and S1B).

**Figure 2:**
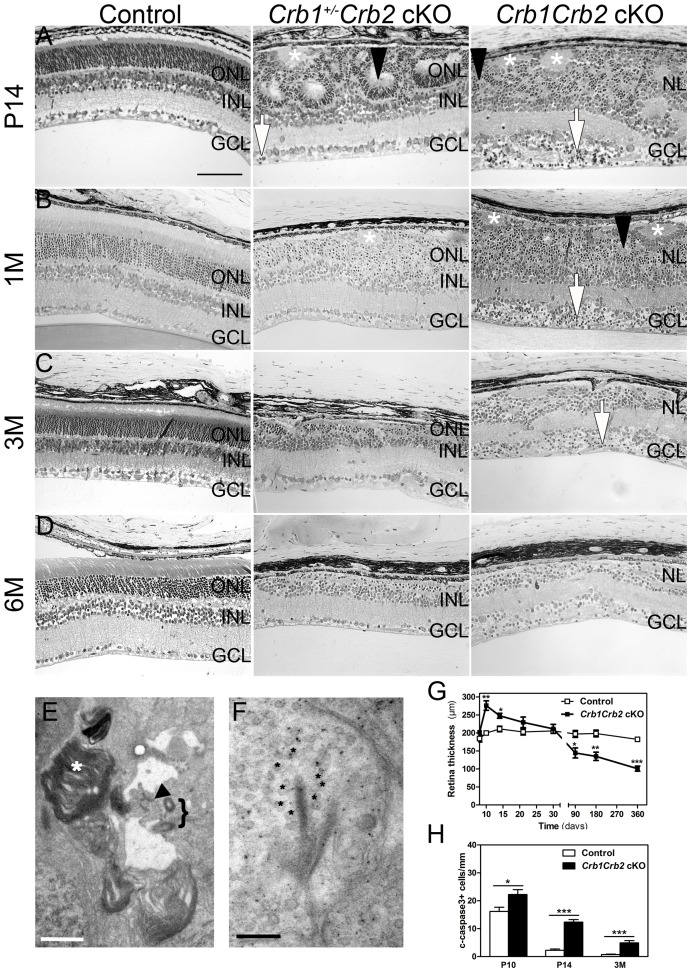
Abnormal layering in *Crb1Crb2* cKO retinas. Histological sections of P14 (A), 1M (B), 3M (C) and 6M (D) old control (left; *Crb1^+/−^Crb2^F/+^* cKO), *Crb1^+/−^Crb2* cKO (middle) and *Crb1Crb2* cKO (right). *Crb1Crb2* cKO retinas had a thick ganglion cell layer and a second broad nuclear layer separated by the inner plexiform layer. *Crb1^+/−^Crb2* cKO had perturbed outer and inner nuclear layers. Ectopic localization of dark-pigmented photoreceptors (white arrows), ganglion/inner nuclear layer cells (white asterisks) and rosettes of photoreceptors (black arrowheads) was visible in the two mutant retinas (Figure S5B,D). Both mutant retinas degenerated rapidly with age. GCL, ganglion cell layer; INL, inner nuclear layer; ONL, outer nuclear layer; (E,F) Electron microscopic pictures of 1M old *Crb1Crb2* cKO retinas. Some complete segments (E, white asterix), adherens junctions (E, black arrow) and centrioles of cilium (E, bracket) or ribbon synapses with vesicles on the two sides of the cleft (F, black asterix) were identified but in ectopic locations. (G) The thickness of 4–5 control and *Crb1Crb2* cKO retinas from P8 to P360. *Crb1Crb2* cKO retinas had a thicker retina than littermate controls at P10 and P14, followed by progressive thinning and degeneration. (H) The cleaved caspase 3 positive apoptotic cells were counted at P10, P14 and 3M from 20–30 sections of 3 littermate controls and *Crb1Crb2* cKO whole retinas. Mutant retinas showed an increase in the number of apoptotic cells. Data are presented as mean ± s.e.m. *P<0.05; **P<0.01; ***P<0.001. Scale bar, 100 µm (A–D); 1 µm (E,F).

**Figure 3:**
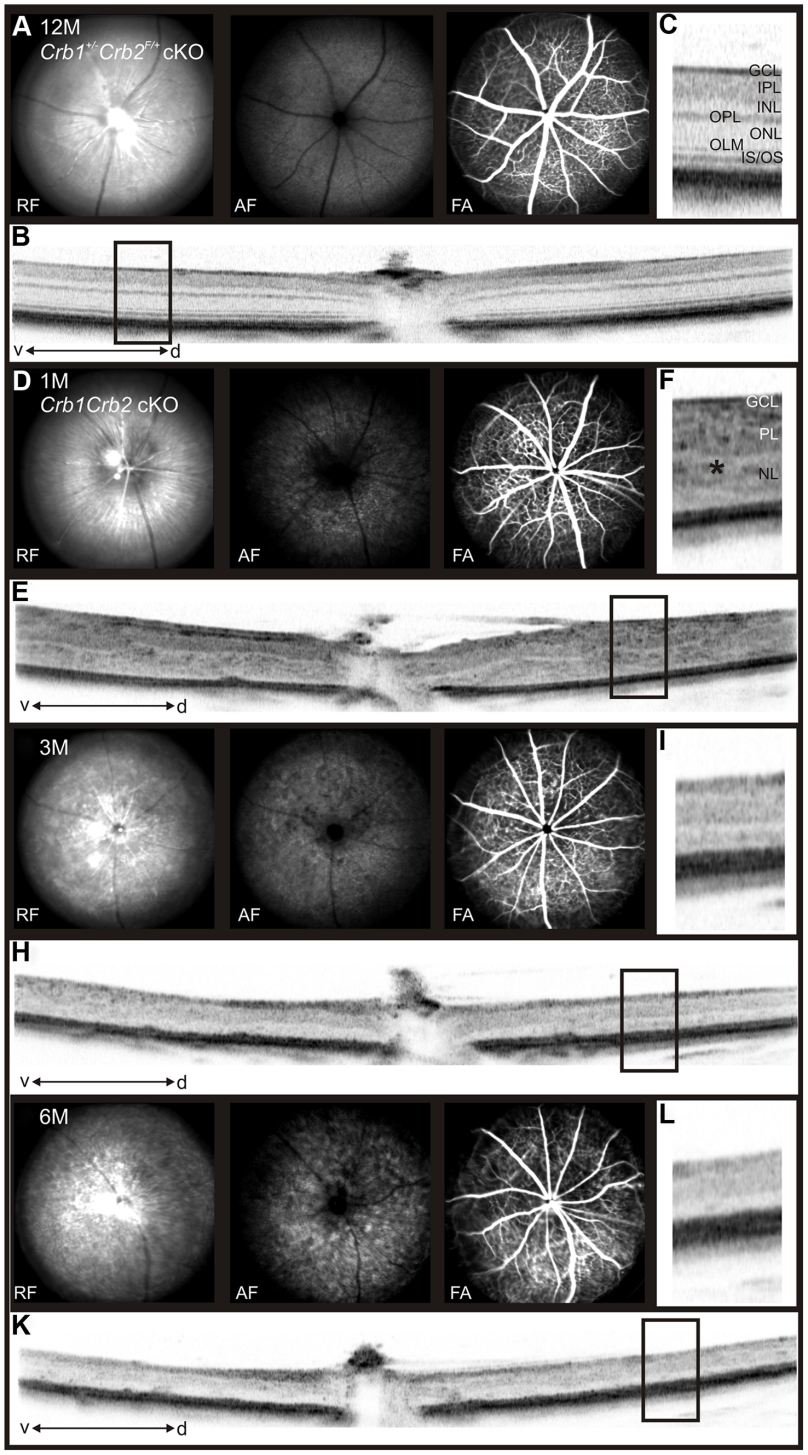
*In vivo* retinal imaging in *Crb1Crb2* cKO mice. 12M old control *Crb1^+/−^Crb2^F/+^* cKO (A–C) and 1M (D–F), 3M (G–I) and 6M (J–L) old *Crb1Crb2* cKO mice were subjected to scanning laser ophthalmoscopy (A,D,G,J) and vertical spectral domain optical coherence tomography (B, E, H, K). C, F, I and L are magnifications of the boxes in B, E, H, and K respectively. At 1M, a disorganized retina with an abnormally thick ganglion cell layer, one plexiform and one nuclear layer was observed (E, asterisk in F). At 3M (G–I) and 6M (J–L), an obvious loss in the retinal thickness was ascertained as well as several fundus alterations (G,J). Abbreviations: AF, autofluorescence; d, dorsal; FA, Fluorescein angiography; GCL, ganglion cell layer; INL, inner nuclear layer; IPL, inner plexiform layer; IS/OS, inner segment/outer segment border; OLM, outer limiting membrane; ONL, outer nuclear layer; OPL, outer plexiform layer; RF, Red-free; v, ventral.

*In vivo* imaging analysis revealed changes in *Crb1^+/−^Crb2* cKO retinas in fundus appearance as well as in retinal layer morphology in contrast to *Crb1^+/−^Crb2^F/+^* cKO control retinas (Figure S2). With native scanning laser ophthalmoscopy, many spots and patchy areas were visible throughout the retina, corresponding to pseudo-rosettes in the photoreceptor layer and in histological sections (Figures S2B and 2A–B). Already at 1M, spectral domain optical coherence tomography revealed an aberrant layering in *Crb1Crb2* cKO retinas (Figure 3E–F). The retina consisted of a single inner plexiform layer, an abnormal thick ganglion cell layer and a second broad nuclear layer (Figure 2A–B). All retinal cell types appeared to be generated, but a separate photoreceptor nuclear layer, inner and outer segment layer and outer plexiform layer were not formed. Two types of rosettes in the broad nuclear layer could be identified and were primarily formed of photoreceptors or ganglion cells and inner nuclear layer cells (Figure 2A–B black arrowheads and asterisks, respectively). Using electron microscopy and immunohistochemistry, we found ectopically localized photoreceptor outer segments, delocalized basal bodies of cilia, adherens junctions and ribbon synapses in the *Crb1Crb2* cKO at 1M (Figures 2E–F and S3A,C).

The retina thickness in the *Crb1Crb2* cKO was significantly increased compared to control retinas at P10 (276.1±13.2 µm vs 199.7±5.4 µm, respectively) and P14 (247.8±6.9 µm vs 211±7.7 µm, respectively; Figure 2G). Both *Crb1Crb2* cKO and *Crb1^+/−^Crb2* cKO retinas degenerate rapidly after 1M, which was associated with retinal vasculature defects leading to the thinning of the retinas in 3–6M retinas (Figures 2C–D, S2 and 3). Quantification of cleaved caspase 3 positive cells showed an increase in the number of apoptotic cells in *Crb1Crb2* cKO retinas at P10, P14 and 3M (Figure 2H). Cleaved caspase 3 positive cells at P10 and P14 were identified as rod photoreceptor cells and at 3M mainly as bipolar cells (Figure S3E–F).

### *CRB1* and CRB2 are essential for proper retinal development

**Figure 4:**
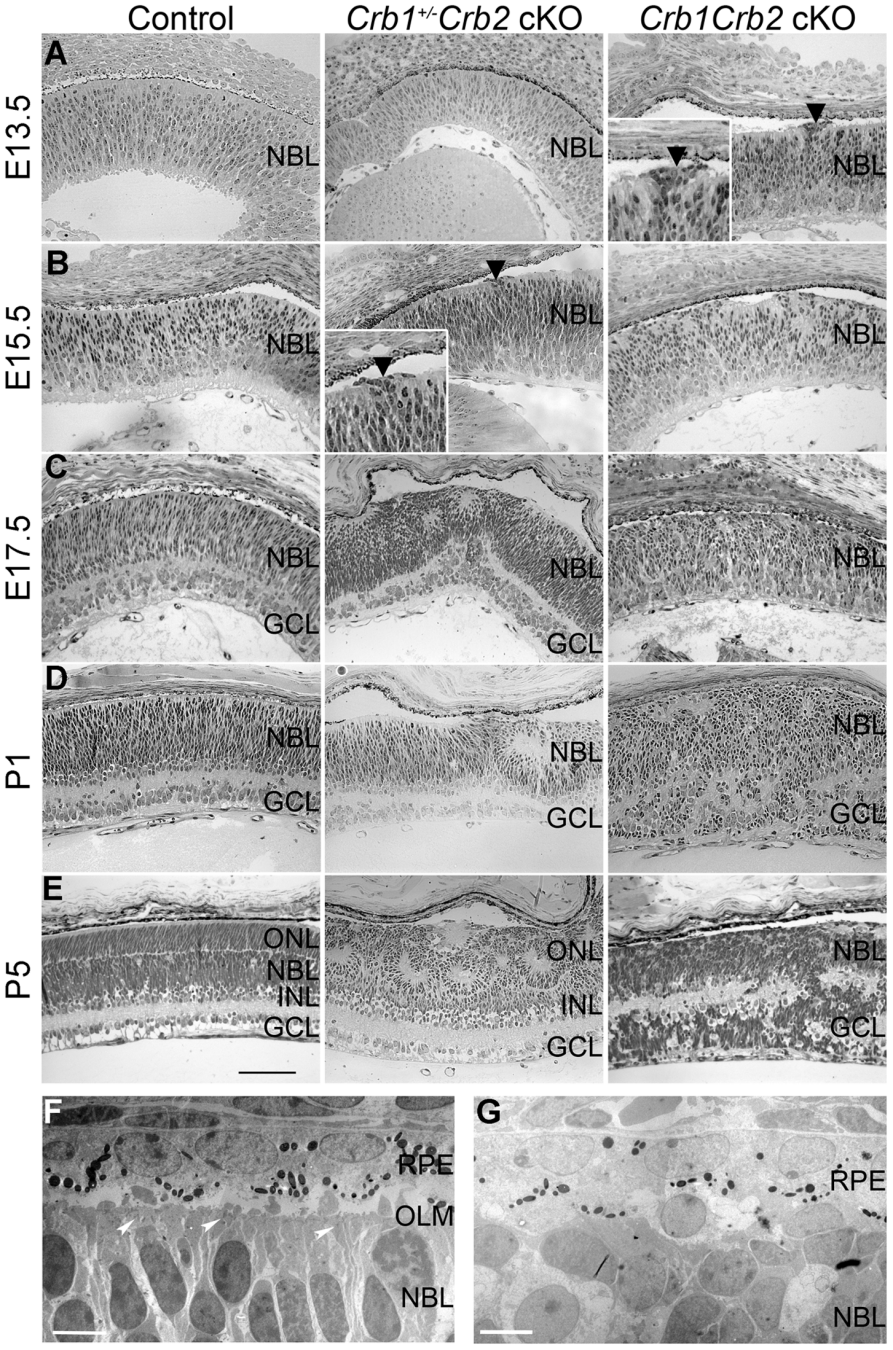
Retinal development is impaired in *Crb1Crb2* cKO. (A–E) Histological sections from E13.5 to P5 control (left), *Crb1^+/−^Crb2* cKO (middle) and *Crb1Crb2* cKO (right). From E13.5 onwards, disruption of the outer limiting membrane (A right, black arrowhead) accompanied with ectopic localization of cells extended in *Crb1Crb2* cKO developing retinas (A,B right). At E17.5 and P1, in contrast to control retinas no proper ganglion cell layer was formed (C,D). The separation of the outer nuclear/photoreceptor layer formed around P5, which never happened in the *Crb1Crb2* cKO retinas (E). *Crb1^+/−^Crb2* cKO retinas showed the first disruption in the outer limiting membrane at the periphery at E15.5 (B middle, black arrowhead), which progressively extended to the centre accompanied with rosette formation (B–E middle). Electron microscopic pictures from E17.5 littermate control (F) and *Crb1Crb2* cKO (G) retinas. Control retinas showed an organized outer limiting membrane with adherens junctions (white arrowheads), retinal pigment epithelium and retinal nuclei alignments. *Crb1Crb2* cKO retinas showed absence of layer organization and adherens junctions. GCL, ganglion cell layer; INL, inner nuclear layer; NBL, neuroblast layer; ONL, outer nuclear layer; RPE, retinal pigmented epithelium. Scale bar, 100 µm (A–E); 5 µm (F,G).

As CRB1 and CRB2 are expressed in the retinal progenitor cells from E12.5 onwards at the subapical region adjacent to adherens junctions [21]–[22] and due to the severe disorganization of these retinas in adult, we analyzed control, *Crb1^+/−^Crb2* cKO and *Crb1Crb2* cKO mice from E11.5 to P5. Whereas no visible defects were observed at E11.5 and E12.5, perturbations at the outer limiting membrane and cellular mislocalizations near the retinal pigment epithelium were visible at E13.5 in *Crb1Crb2* cKO retinas (Figure 4A, black arrowhead). Between E15.5 and E17.5 in *Crb1Crb2* cKO, the adherens junctions were gradually lost and the nuclei of the retinal progenitors showed abnormal orientation, whereas in control retinas, progenitors were arranged radially along the apical-basal axis (Figures 4B–C and S4B). Electron microscopic analyses showed loss of adherens junctions in the neural retina and ectopic nuclei close to the retinal pigment epithelium (Figures 4F–G and S4E–F). During retinogenesis, the photoreceptor layer and the outer plexiform layer formed at P5. However, in the *Crb1Crb2* cKO, this process never ensued, as no distinct photoreceptor layer was formed (Figure 4E).

In *Crb1^+/−^Crb2* cKO, perturbations at the outer limiting membrane started at the periphery of the retina at E15.5 (Figure 4B, black arrowhead). It progressively extended to the centre of the retinas where rosettes also formed (Figure 4C–E). In late developmental stages, in addition to photoreceptor rosettes, ganglion cell nuclei and inner nuclear layer cells were found in the outer nuclear layer and some photoreceptor nuclei were found in the ganglion cell layer (Figure 2A). These retinas display intermediate phenotypes between the *Crb2* cKO [22] and *Crb1Crb2* cKO.

### Increased number and mislocalization of late born cells in *Crb1Crb2* cKO retinas

**Figure 5:**
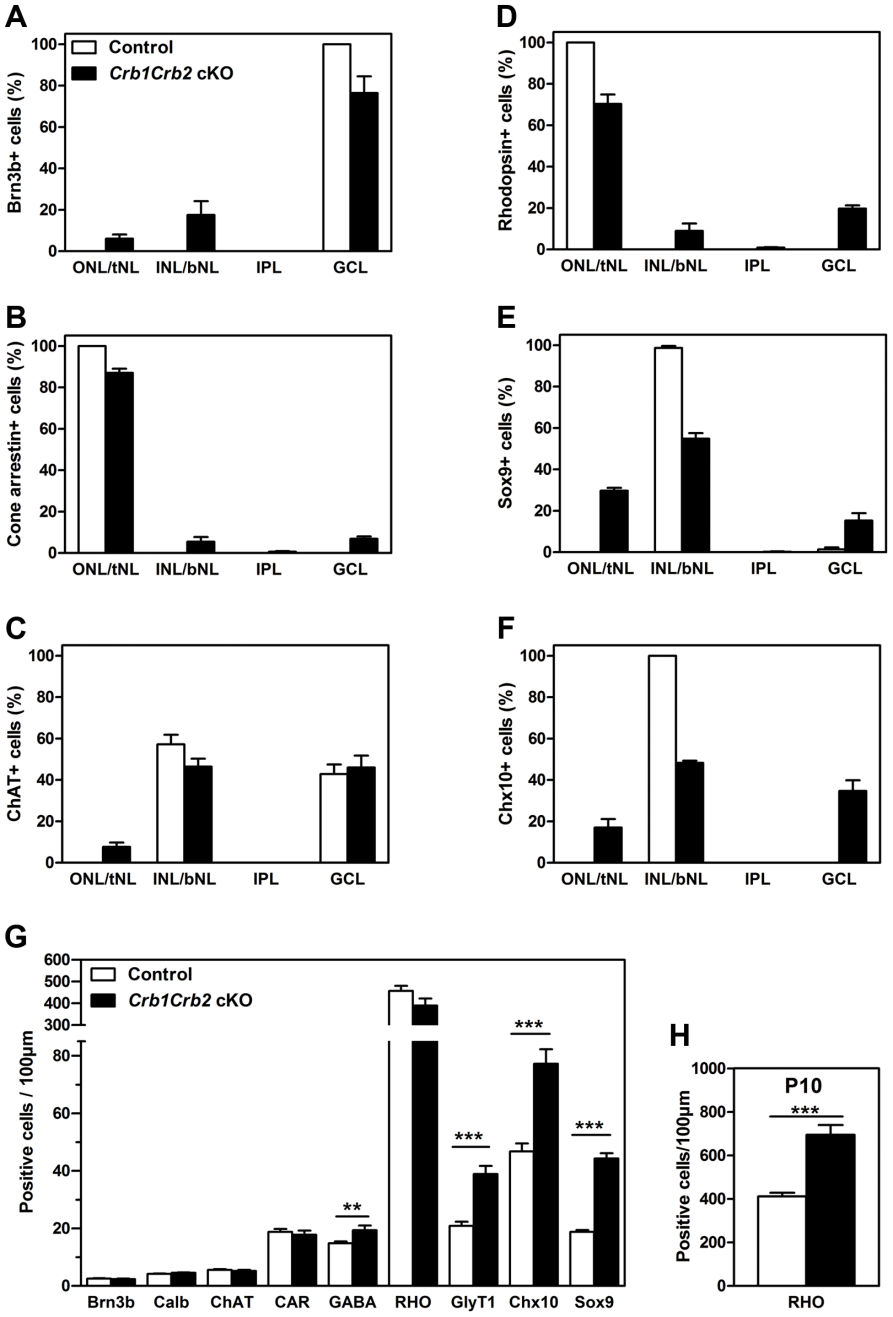
The number of late born cell types is increased in *Crb1Crb2* cKO retinas. The distribution of early (A–C) and late born (D–F) cell types in the three layers was quantified as a percentage of each cell type in outer, inner and ganglion cell nuclear layer in the control (white bars), and top and bottom half of nuclear layer (tNL and bNL) and ganglion cell layer in the *Crb1Crb2* cKO (black bars) retinas at P14 (3–4 different animals/genotype). The distribution of early-born ganglion cells (Brn3b), cone photoreceptors (cone arrestin) and cholinergic amacrine cells (choline acetyltransferase, ChAT) was slightly affected in contrast to late-born rod photoreceptors (rhodopsin), Müller cells (Sox9) and bipolar cells (Chx10), which were to a larger extent wrongly distributed in the two nuclear layers. (G,H) The number of cells for each cell types was quantified at P14 (G) and the rods at P10 (H) in 3–4 retinas of control and *Crb1Crb2* cKO, and represented by the mean ± s.e.m. The number of early born cells was not affected whereas the number of late born cells was increased in *Crb1Crb2* cKO compared to control retinas at P14 and the rods at P10. bNL, bottom nuclear layer; Calb, calbindin positive horizontal cells; CAR, cone arrestin; GCL, ganglion cell layer; GlyT1, glycinergic amacrine cells; INL, inner nuclear layer; IPL, Inner plexiform layer; ONL, outer nuclear layer; RHO, Rhodopsin; tNL, top nuclear layer. **P<0.01; ***P<0.001.

Due to the severe disorganisation of the retinas, we further investigated whether all retinal cell types formed in the absence of CRB1 and CRB2. Using specific markers for the different cell types, we found that all the different cell types formed and there were no indications for hybrid retinal cell types (Figure S5 and data not shown). Several of the retinal cell types appeared to localize ectopically. To further analyze this, we compared the localization of the cell nuclei in the top and bottom parts of the broad nuclear layer in *Crb1Crb2* cKO mice to the outer and inner nuclear layer in control retinas (Figures 5A–F and S5A–F). The localization of the earliest born cell types, ganglion cells (marked by Brn3b), cone photoreceptors (Cone arrestin), horizontal cells (Calbindin) and the earliest born amacrine cells (ChAT) was less affected than the late born cell types, rod photoreceptors (Rhodopsin), Müller cells (Sox9 and glutamine synthetase) and bipolar cells (PKCα or *Cre-GFP* under the Chx10 promoter). In *Crb1^+/−^Crb2* cKO retinas, rods, cones and bipolar cells localized ectopically in the ganglion cell layer (Figure S5G–H), and amacrine and ganglion cells surrounded by bipolar cells formed pseudo-rosettes in the photoreceptor layer (Figure S5I–J). These results suggest that all cell types are generated in retinas that lack CRB1 and CRB2 but their normal migration/localization is affected.

To test whether retinal cell types formed in normal numbers, we counted the different cell types at P14 (Figure 5G). The number of early born cells was unchanged whereas the number of late born cells was increased compared to control retinas: GABAergic amacrine cells (19.4±1.6 versus 14.8±0.6 cells/100 µm), late born GlyT1 positive amacrine (38.9±2.8 versus 20.9±1.4 cells/100 µm), Chx10^+^ bipolar cells (77.2±5.0 versus 46.7±2.8 cells/100 µm) and Sox9^+^ Müller cells (44.3±1.8 versus 18.8±0.6 cells/100 µm). At P14, the number of rod photoreceptors was not significantly increased due to ongoing apoptosis (Figures 3H and S3E). We found at P10 an increase in number of rods (695±44 in *Crb1Crb2* cKO and 412±17 cells/100 µm in control; Figure 5H). This finding suggests that CRB1 and CRB2 may play a role in regulating the proliferation of the retinal progenitors.

### Increased cell proliferation and apoptosis in developing *Crb1Crb2* cKO retinas

**Figure 6:**
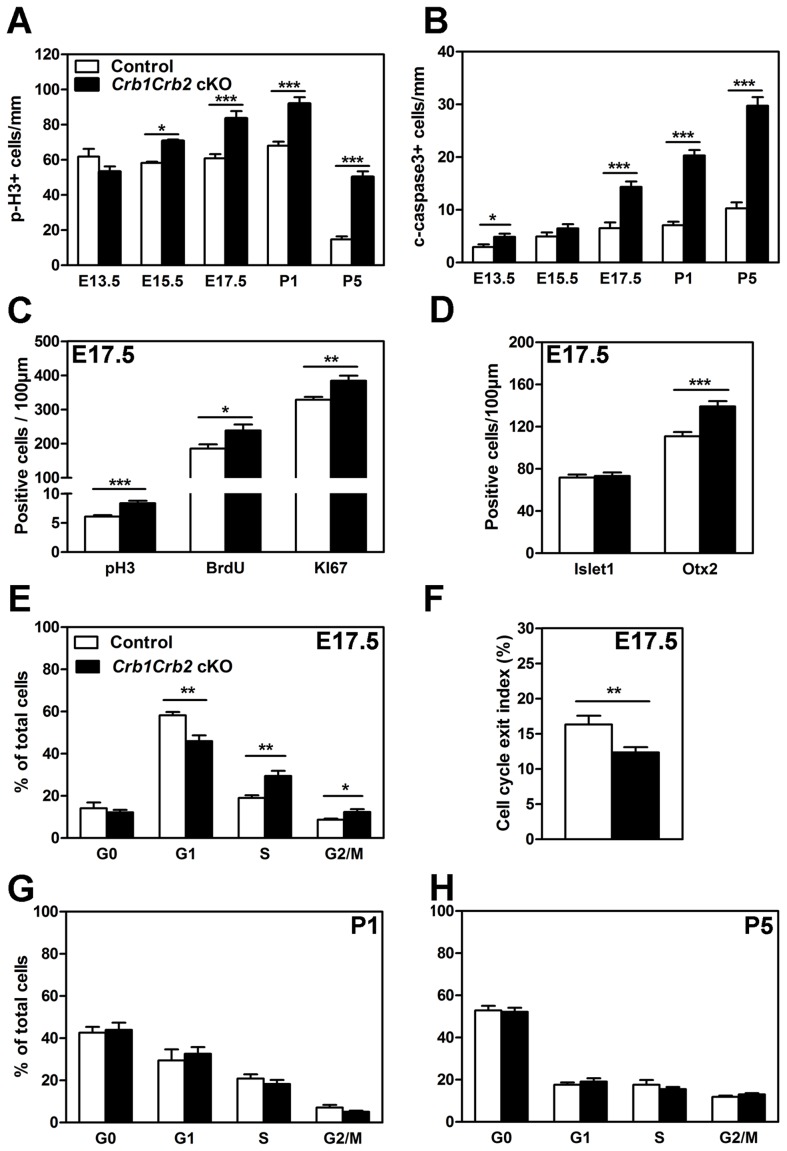
Loss of CRB1 and CRB2 leads to cell cycle defects, increased proliferation and apoptosis. The number of mitotic cells immunostained with anti-phospho-Histone H3 (pH3; A) and apoptotic cells immunostained with cleaved caspase 3 (B) were quantified from E13.5 to P5. *Crb1Crb2* cKO retinas showed a significant increased number of mitotic and apoptotic cells from E15.5 and E17.5 respectively in comparison to control retinas. At E17.5, the number of mitotic cells in the cell cycle using pH3 (M-phase), 30 min-pulse BrdU labelling (S-phase) and Ki67 immunostaining (all phases) was increased in *Crb1Crb2* cKO retinas (C). Quantification at E17.5 showed that the number of early-born (Islet1, amacrine and ganglion cells) progenitor cells was not affected whereas late-born (Otx2, photoreceptors) progenitor cells was increased in *Crb1Crb2* cKO compared to control retinas (D). Cell cycle exit index (F) was determined as the ratio of BrdU^+^/Ki67^−^ cells (no longer dividing) to total (24 hours) BrdU^+^ cells. In *Crb1Crb2* cKO retinas less cells exit the cell cycle in the BrdU labelled population. Data from 20 representative sections/pictures of whole retinas from 3–5 control and *Crb1Crb2* cKO retinas are presented as mean ± s.e.m. Flow cytometry analysis of cell cycle in *Crb1Crb2* cKO and control retinas at E17.5 (E), P1 (G) and P5 (H) revealed that only at E17.5 the proportion of cells in the different cell cycle phases is changed compared to control. *P<0.05; **P<0.01; ***P<0.001.

In the *Crb1Crb2* cKO retinas, the increased number of late born cells might be due to overproliferation of progenitors or reduced apoptosis. Therefore, in control, *Crb1^+/−^Crb2* cKO and *Crb1Crb2* cKO retinas from E13.5 to P5 animals, we analysed the number of phospho-Histone H3 (pH3) positive cells and cleaved caspase 3 positive cells, which are markers for mitotic cells and apoptotic cells respectively (Figures 6A–B and S6C–D). From E15.5 onwards, the number of M-phase cells was significantly increased in *Crb1Crb2* cKO retinas, and the number of apoptotic cells was increased at E13.5 and E17.5 onwards. These data showed an increase in both mitosis and apoptosis in retinas lacking CRB1 and CRB2. Furthermore, cells in M-phase are normally located at the apical region in control retinas. However, in E17.5 *Crb1Crb2* cKO retinas, where the apical region was almost completely lost, the cells in M-phase localized randomly throughout the entire thickness of the retina (Figure S6C–D). To test whether precursor cells formed in normal numbers, we counted at E17.5 early and late-born precursor cells. The number of Islet1^+^ early-born precursor cells (ganglion and amacrine cells) is unchanged in contrast to an increased number of Otx2^+^ late-born precursor cells (photoreceptors and bipolar cells; 139.3±5 cells/100 µm in *Crb1Crb2* cKO retinas versus 110.9±4.1 cells/100 µm in control; Figure 6D).

At E17.5, in *Crb1^+/−^Crb2* cKO retinas, the number of mitotic and apoptotic cells was increased like in *Crb1Crb2* cKO retinas (Figure S6A–B). However, at P5 an increased number of mitotic cells and a decreased number of apoptotic cells were observed like in *Crb2* cKO [22], indicating that the *Crb1^+/−^Crb2* cKO showed intermediate features between *Crb2* and *Crb1Crb2* cKO.

### Dysregulation of the cell cycle in *Crb1Crb2* cKO retinas

We further investigated, at E17.5, which phases of the cell cycle were affected using a combination of 30 min pulse labelling with BrdU for the S-phase, phospho-Histone H3 (pH3) for the M-phase and Ki67 labelling, a marker for M, G2, S and late G1 phases of the cell cycle (Figures 6C, S6C and S6E). This showed that in *Crb1Crb2* cKO retinas the number of pH3^+^ (6.1±0.2 in control versus 8.4±0.4 cells/100 µm in cKO retinas), BrdU^+^ (185.9±12.1 in control versus 238.8±17.5 cells/100 µm in cKO retinas) and Ki67^+^ cells (329±8.3 in control versus 384.5±15 cells/100 µm in cKO retinas) were increased. In mice, the proportion of dividing cells decreases dramatically at the centre of the retinas from P5 onwards, whereas the progenitors at the periphery of the retina still proliferate. Ultimately, mitosis is finished at the centre at P6 and at the periphery at P10 [3]. Surprisingly, in *Crb1Crb2* cKO retinas the number of cells in M-phase (pH3^+^) was higher compared to the controls (Figure 6A). We further investigated this phenomenon using the Ki67 marker to analyse the proliferating cells in all phases of the cell cycle (Figure S6D,F) and found that the total number of cells was increased by a factor of two both in the centre and at the periphery at P5 (Figure S6H). In contrast to the control, some Ki67 positive cells were still present at the periphery of the retina at P10 in *Crb1Crb2* cKO retinas (data not shown). These results suggest that active proliferating cells in *Crb1Crb2* cKO retinas may reside longer than those in control retinas.

**Figure 7:**
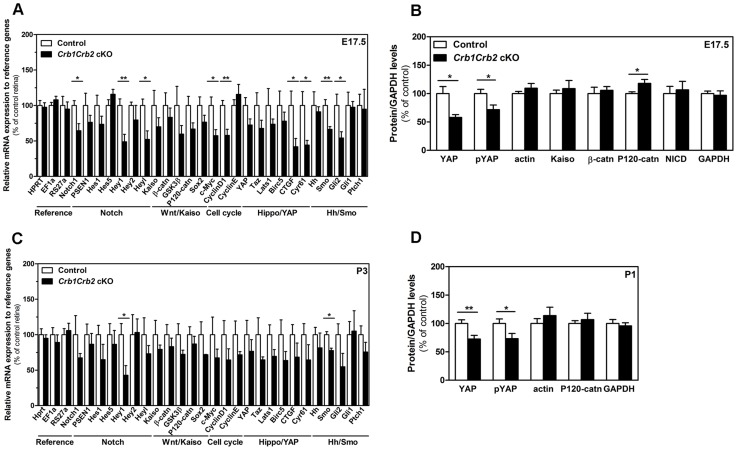
CRB1 and CRB2 acts on the proliferative signalling pathways. Transcript levels measured by quantitative PCR at E17.5 (A) and P3 (C) in 3–6 control and *Crb1Crb2* cKO retinas showed changes in Notch1, YAP, sonic hedgehogs and cell cycle genes at E17,5 whereas at P1 these genes were not significantly changed except Hey1 and Smoothened. Quantification of protein levels of control and *Crb1Crb2* cKO retinal lysates (N = 3–5 for each Western blot and Western blots were repeated 2–4 times) at E17.5 (B) and P1 (D). Protein levels of YAP and pYAP were reduced at E17.5 and P1 whereas P120-catenin was increased and β-catenin and Kaiso unchanged at E17.5. Data are presented as mean ± s.e.m *P<0.05; **P<0.01.

We performed flow cytometry analysis based on the DNA content and KI67 labelling at E17.5, P1 and P5 to study the proportion of cells at G1, S and G2/M phases of the cell cycle or which already exited the cell cycle in G0 (Figures 6E, G, H and S6G). At E17.5, the proportion of cells in G1 was reduced whereas the proportion of cells in S and G2/M was increased and G0 unchanged. At P1 and P5, the proportion of cells in *Crb1Crb2* cKO returned to control proportion. In addition, levels of *cyclin D1*, cyclin *E* and *c-myc* transcripts (Figure 7A) were changed suggesting also an aberrant regulation of the cell cycle in *Crb1Crb2* cKO retinas at E17.5.

We examined how the cell cycle exit was affected in the mutants by injecting BrdU at E16.5 and analysing 24 hours later (Figures 6F and S6F) [29]–[30]. The proportion of cells which exit the cell cycle (BrdU^+^KI67^−^) in the total population of BrdU labelled cells was significantly decreased in *Crb1Crb2* cKO retinas (12.3±0.7%) compared to control (16.3±1.3%). However, the number of BrdU^+^KI67^−^ cells per 100 µm is not significantly different (40.2±2.9) compared to control (35.3±1.8). In summary, our data suggest that the increased population of late progenitor cells and late born cells is due to dysregulation of the cell cycle at E17.5.

### *CRB1* and CRB2 restrain the overproliferation of the progenitors via the regulation of mitogenic signaling pathways

We investigated which proliferative signalling pathway(s) might be involved in the overproliferation of the murine progenitors in *Crb1Crb2* cKO retinas at E17.5 and in early postnatal days.

The phospho-S6 ribosomal protein (pS6RB), a downstream target of mTOR signalling, localised in the post-mitotic cells in the retina and the number of the pS6RB positive cells or pS6RB protein levels at E17.5 and P1 were unchanged in *Crb1Crb2* cKO retinas, suggesting that mTOR signalling is not affected in the retina upon removal of CRB1 and CRB2 (Figure S7D and data not shown).

No differences were observed in the primary downstream targets Gli1 and Ptch1 of sonic hedgehog signalling (Figure 7A). The downregulation of Smoothened and Gli2 might be due to a secondary effect of the loss of CRB proteins. The sonic hedgehog signalling seemed to not be directly involved in the increased number of progenitors.

In E17.5 and P1 retinas, whereas no difference in the amount of cleaved active intracellular form of Notch1 protein was detected, the transcript levels of Notch1 and its primary downstream targets Hey1 and Heyl were reduced in *Crb1Crb2* cKO compared to control (Figures 7 and S7). The Notch1 signalling might be affected following loss of CRB1 and CRB2.

The role of Wnt-β-catenin canonical signalling in retinal proliferation remains controversial. In E17.5 control retinas, P120-catenin and β-catenin localized mainly in the adherens junctions at the subapical region whereas in the *Crb1Crb2* cKO the adherens junctions were disrupted and the catenins are membrane-associated (Figure S7A–B, white arrowheads). At E17.5, levels of P120-catenin proteins were increased in *Crb1Crb2* cKO retinas, in contrast to β-catenin, whereas transcript levels were unchanged (Figures 7 and S7E–F). Furthermore, we showed that the zinc finger protein Kaiso was expressed in E17.5 and P1 developing retinas, but that its protein levels were not affected in *Crb1Crb2* cKO mice (Figures 7 and S7E). The presence of Kaiso in the retina and the increased levels of P120-catenin proteins are of interest as the inhibition of Kaiso on Wnt signalling is blocked through its interaction with P120-catenin (Figure 7E) [31], [32].

Only recently, YAP, the downstream effector of the Hippo pathway, has been reported to promote the proliferation of the murine progenitors in postnatal retinas, followed by downregulation around P5 during neuronal differentiation [33]. In control mice, YAP protein was detected in progenitor nuclei, overlapping with Chx10*Cre-GFP* localization (Figure S7C). YAP localized also at the apical region where the adherens junctions and the CRB complex were located. In the *Crb1Crb2* cKO retinas, YAP localized at the remaining subapical region and only in the cytoplasm of the progenitors (Figure S7C). Phosphorylation of YAP causes its retention in the cytoplasm and binding to the adherens junctions, thus inactivating the protein [14]. Both YAP and phospho-YAP (pYAP) protein levels and the transcripts of the direct downstream targets genes CTGF and Cyr61 were reduced in *Crb1Crb2* cKO retinas at E17.5 and P1 (Figure 7B,D). The YAP signalling is affected by the loss of CRB1 and CRB2.

## Discussion

One key element in the construction of the retina during development is the tight control of the proliferation and differentiation of the retinal progenitor cells by a combination of extrinsic and intrinsic influences [2]. In this study, we analyzed the effect of ablation of CRB1 and CRB2 in the murine retina and showed that levels of CRB protein control the lamination and proliferation of the progenitors. Complete loss of CRB1 and CRB2 proteins in the mouse retina mimics human LCA due to mutations in the *CRB1* gene.

The adherens junctions play a critical role in the migration of post-mitotic cells from the apical surface to their final destination [34]. Ganglion, bipolar and photoreceptor cells extend basal processes that guide nucleus translocation to their final destination. Bipolar and ganglion cells relinquish their apical attachment when translocation is complete whereas photoreceptors maintain adherens junctions with Müller glial cells. Amacrine and horizontal cells by contrast display active cellular migration without apical attachment by sensing their local environment [34]. Disruption of the apical adherens junctions/subapical region in *Crb1^+/−^Crb2* cKO retinas at E15.5 leads to ectopic localization of some photoreceptor and bipolar cells in the ganglion cell layer and vice versa, ganglion, amacrine and bipolar cells in the outer nuclear layer. In *Crb1Crb2* cKO mice, where the disruption occurs two days earlier, the lack of apico-basal axis leads to distribution of all the cell types in two nuclear layers and lack of a separate photoreceptor layer. Photoreceptor, ganglion and bipolar cells may undergo misguided migration due to the lack of apical attachment. The localization of the earliest born cells remains less affected, probably due to completion of migration prior to adherens junction disruption.

Apart from the role in orchestration of migration, we suggest a direct role of CRB proteins in regulation of proliferation of retinal progenitors. *Crb1* KO retinas do not show an obvious developmental phenotype [24], and *Crb2* cKO retinas show an increase in the number of progenitors only at P3 [22]. However, the *Crb1Crb2* cKO showed increased number of mitotic cells from E15.5 to P10 and *Crb1^+/−^Crb2* cKO retinas at E17.5 and P5. Thus, the uncontrolled proliferation of progenitors is proportional to the lack of CRB1 and CRB2 proteins. A study on CRB-interacting protein PALS1 has shown that the CRB complex might be involved in the control of progenitor proliferation in the developing mouse cortex [9]. However, in mouse retinas, conditional knockout or knockdown of *Pals1* does not lead to increased proliferation of retinal progenitor cells [35], [36]. The role of CRB protein on the proliferation of the progenitors may be independent of PALS1 and involve other partners. Ablation of CRB1 and CRB2 proteins leads to an increased number of proliferating cells and abnormalities in the cell cycle. Hence, CRB proteins restrain the proliferation acting on the cell cycle machinery. Additionally, the lack of the apical CRB1 and CRB2 had an effect on the cell cycle exit potentially directing the decision to re-enter the cell cycle and explaining the increased number of progenitors. The reduced number of cells withdrawing the cell cycle may explain why retinal progenitor cells in *Crb1Crb2* cKO retinas undergo several more cell cycles compared to control retinas, leading to an increase in number of late-born cell types and significant thickening of the retina.

Here, we report that CRB1 and CRB2 act on the proliferation of the retinal progenitor cells through dysregulation of the proliferative signalling pathways such as Notch1 and YAP/Hippo. In addition, we report the presence of Kaiso in the retina and increased level of P120-catenin at E17.5. We hypothesize that the lack of CRB1 and CRB2 leads to disruption of the adherens junction complex and release of available β- and P120-catenins in the cytoplasm and nuclei of progenitors. P120-catenin may retain Kaiso in the cytoplasm leading to the loss of inhibition of the Wnt target genes. Overexpression of P120-catenin and Kaiso has been linked to aberrant mitosis in cancer cells [37], [38]. Lack of CRB proteins affects the YAP/Hippo pathway. Despite its direct role on proliferation, YAP promotes cell survival by inhibiting apoptotic pathways [14]. The decrease in YAP signalling at E17.5 and P1 in *Crb1Crb2* cKO might explain the increase in apoptosis observed.

Mutations in the *CRB1* gene cause progressive autosomal-recessive retinitis pigmentosa and LCA. *CRB1*-LCA retinas are remarkably thick and lack distinct layers as detected by optical coherence tomography [20]. Mice lacking CRB1 function show limited and mild retinal disorganization in the inferior quadrant [24]–[27]. Prominent differences were found between the severe loss of retinal function in humans and the mild phenotype in mice [39]. In contrast, *Crb2* cKO mice display a severe phenotype with progressive loss of photoreceptors and retinal activity mimicking *CRB1*-related retinitis pigmentosa [22]. Many genes involved in retinal dystrophies have been reported to show difference in temporal and spatial expression patterns and in their localization inside the retina [40], [41]. Furthermore, compensation by other members of the same protein family occurs frequently in mice and humans such as the tumor suppressor genes during retinal development [42]. Further investigations on CRB1 and CRB2 would be needed to completely understand the difference between mice and humans.

From *Crb2* cKO, *Crb1^+/−^Crb2* cKO and *Crb1Crb2* cKO retina studies, the severity of the retinal disease is inversely proportional to the amount of CRB1 and CRB2 proteins which seemed to be critical for the development of the retina. As no genotype-phenotype correlation in *CRB1* retinal dystrophies has been identified [43], additional down-regulation of CRB2 function in human *CRB1*-mutant retinas might range from *CRB1*-retinitis pigmentosa to *CRB1*-LCA. Several polymorphisms in highly conserved residues have been identified in the *CRB2* gene but not directly linked to retinal dystrophies [44]. Further investigations on possible mutations in CRB complex member genes in *CRB1*-LCA versus *CRB1*-RP patients might address the question of the genotype-phenotype correlation.

Here, we report that *Crb1Crb2* retinas display a thicker retina due to excessive proliferation of late-born retinal progenitor cells and also immature layering. Moreover, *Crb1Crb2* and *Crb1^+/−^Crb2* cKO animals show severe loss of retinal function. *Crb1Crb2* and *Crb1^+/−^Crb2* cKO retinas exhibit the characteristics of human *CRB1*-LCA retinopathies, and are therefore mouse LCA models for the development of therapeutic drugs.

## Materials and Methods

### Animals

Animal care and use of mice was in accordance with protocols approved by the Animal Care and Use Committee of the Royal Netherlands Academy of Arts and Sciences (KNAW). All mice used were maintained on a 50% C57BL/6JOlaHsd and 50% 129/Ola genetic background. Animals were maintained on a 12 h dark/dim light cycle and supplied with food and water *ad libitum*. *Crb1* KO mice [24] and *Crb2^F/F^*Chx10*Cre^Tg/+^* clone P1E9 (*Crb2* cKO) generated previously [22] were crossed to generate *Crb1^+/−^Crb2^F/F^*Chx10*Cre^Tg/+^* (*Crb1^+/−^Crb2* cKO) and *Crb1^−/−^Crb2^F/F^*Chx10*Cre^Tg/+^* (*Crb1Crb2* cKO). *Crb1Crb2* cKO retinas were compared to littermate *Crb1^−/−^Crb2^F/F^* and *Crb1^−/−^Crb2^F/+^*Chx10*Cre^Tg/+^* retinas and *Crb1^+/−^Crb2* cKO to littermate *Crb1^+/−^Crb2^F/+^* cKO. Chromosomal DNA isolation and genotyping were performed as previously described [22].

### *In vivo* analysis

Scanning laser ophthalmoscopy (SLO), spectral domain optical coherence tomography (SD-OCT) and electroretinography (ERG) measurements were performed at 1, 3, 6 and 12 month in 4 to 6 animals of each genotype. Electroretinograms were recorded binocularly as described previously [45]. Single-flash responses were obtained under scotopic (dark-adapted overnight) and photopic (light-adapted with a background illumination of 30 cd/m^2^ starting 10 minutes before recording) conditions. Single white-flash stimuli ranged from −4 to 1.5 log cd s/m^2^ under scotopic and from −2 to 1.5 log cd s/m^2^ under photopic conditions. Ten responses were averaged with inter-stimulus intervals of 5 s (for −4 to −0.5 log cd s/m^2^) or 17 s (for 0 to 1.5 log cd s/m^2^). Retinal morphology of the anesthetized animals was visualized via SLO imaging with a HRA 1 and HRA 2 (Heidelberg Engineering, Heidelberg, Germany) according to previously described procedures (Text S1) [46]. SD-OCT imaging was performed with a commercially available Spectralis HRA+OCT device from Heidelberg Engineering. This equipment features a broadband superluminescent diode at λ = 870 nm as low coherent light source (Text S1) [47].

### Morphological analysis

Eyes were collected from embryonic day E11.5 to 12M (n = 3–5/age/group) and were fixed at room temperature with 4% paraformaldehyde in PBS. Eyes were dehydrated in ethanol and embedded in Technovit 7100 (Kulzer, Wehrheim, Germany) and sectioned (3 µm). Slides were dried, counterstained with 0.5% toluidine blue and mounted under coverslips using Entellan (Merk, Darmstadt, Germany). The thickness of the retina in *Crb1Crb2* cKO mice from P8 to 12M was measured from the outer limiting membrane to the inner limiting membrane (from top to bottom of *Crb1Crb2* cKO retinas) at exactly 1 mm apart from the optic nerve and the average of the ventral and dorsal measurement was compared to the dorsal measurement of control mice.

### Standard transmission electron microscopy

E17.5 and 1M old mice were perfused with 4% paraformaldehyde, 2% glutaraldehyde in 0.1 M cacodylate buffer pH 7.4. After the retinas were dissected free, they were post-fixed in 1% osmium tetroxide. Tissues were thoroughly rinsed and stained with 2% uranyl acetate in 70% ethanol. Samples were then dehydrated in a graded series of ethanol and embedded in epon 812 (Polysciences). Ultrathin sections were examined with a Zeiss 912 electron microscope.

### Quantification of apoptotic, proliferating and retinal cells

Positive cells (Table S1) from 20–30 representative sections of the whole retina from 3–5 different control or experimental animals were manually counted and corrected by the length of each section (measured using ImageJ software fiji-win32). Retina sections of E13.5 to P5 were stained with cleaved Caspase 3 (cCasp3; marker for apoptotic cells) and phospho-Histone H3 (pH3; marker for M-phase mitotic cells) antibodies.

To examine the proportion of progenitors in S-phase, pregnant females were injected with BrdU (50 µg/g body weight) at E17.5 and embryos were collected 30 min after BrdU injection. To examine the number of progenitors which exit the cell cycle, pregnant females were injected with BrdU at E16.5 and embryos were collected 24 h later. The number of BrdU^+^Ki67^−^ cells represents the number of cells which have exited the cell cycle. The number of retinal cells at P14 was counted on 20–30 representative pictures of retinas stained with specific antibodies for each cell type. Cones, rods, horizontal, Müller and ganglion cells were counted using cone arrestin, rhodopsin, calbindin, Sox9/glutamine synthetase and Brn3B antibodies, respectively. Bipolar cells were counted using PKCα staining and *Cre-GFP* expression (GFP is fused to the Cre in Chx10*Cre* mouse line). Subsets of amacrine cell types were stained using choline acetyltransferase (ChAT), GABA, and GlyT1 antibodies.

### Flow cytometry

These experiments were performed similarly to [29]. Retinas from at least 4 controls and *Crb1Crb2* cKO were isolated and mechanically dissociated with colagenase/DNAse I (370 U) at 37°C. Cells were fixed with 4% paraformaldehyde in PBS for 5 minutes followed by fixation in ethanol 70% one hour at 4°C. Cells were labelled with KI67 antibody diluted 1/50 in PBS-0.5% Tween-20-BSA 0.1% (PBS-TB) overnight at 4°C followed by goat anti-mouse-Alexa 488 antibody diluted 1/500 in PBS-TB. DNA content was labelled with PBS-TB containing 100 µg/ml RNase A 30 minutes at 37°C followed by 100 µg/ml propidium iodide 30 minutes. Cells analysis was performed using the flow cytometer BD LSR Fortessa. See more details about the analysis in Text S1.

### Western blotting

The E17.5 and P1 retinas from at least 3 *Crb1Crb2* cKO or control littermate mice were isolated, homogenized and incubated on ice in 20 µL of lysis buffer (10% glycerol, 150 mM NaCl, 1 mM EGTA, 0.5% Triton x-100, 1 mM PMSF, 1.5 mM MgCl_2_, 10 µg/µL aprotin, 50 mM Hepes pH 7.4 and protease inhibitor cocktail). Retina extracts from 3 independent control and *Crb1Crb2* cKO animals were fractionated by SDS-PAGE electrophoresis, using 4–12% precast gels (NuPage Novex Bis-Tris Mini Gels, Invitrogen). After transfer to nitrocellulose membrane and blocking in 5% BSA in T-TBS buffer (Tris-HCL 50 mM pH7.5, 200 mM NaCl, 0.05% Tween-20), the primary antibodies (table S1) were diluted 1/1000 in T-TBS-5% BSA and incubated overnight at 4°C. After washing, they were incubated with the appropriate secondary antibodies (conjugated to DyLight Dye-800, Li-COR Odyssey or to cyanine 5) diluted 1/5000 in T-TBS buffer. After washing, the blots were then scanned using LI-COR Odyssey IR Imager. Densitometry of bands was performed in ImageJ. The densitometry for each band was subtracted to the background and normalized with GAPDH densitometry from the same sample.

### Real-time quantitative PCR

RNA was isolated from 3–6 control and *Crb1Crb2* cKO retinas using TRIZOL reagent (Gibco life technologies), according to the manufacturer manual, and after the final precipitation dissolved in RNase-free water. After genomic DNA degradation with RNase-free DNase I (New England Biolabs), 1 µg of total RNA was reverse transcribed into first-strand cDNA with Superscript III Plus RNase H-Reverse Transcriptase (Invitrogen) and 50 ng random hexamer primers, during 50 min at 50°C in a total volume of 20 µl. To the resulting cDNA sample, 14 µl of 10 mM Tris, 1 mM EDTA was added. From all samples, a 1∶20 dilution was made and used for qPCR analysis. For this analysis, primer pairs were designed with a melting temperature of 60–62°C, giving rise to an amplicon of 80–110 bp. Real-time qPCR was based on the real-time monitoring of SYBR Green I dye fluorescence on a ABI Prism 7300 Sequence Detection System (Applied Biosystems, Nieuwekerk a/d IJssel, The Netherlands). The PCR conditions were as follows: 12.5 µL SYBR Green PCR 2× mastermix (Applied Biosystems), 20 pmol of primers, and 2 µl of the diluted cDNA (ca 3 ng total RNA input). An initial step of 50°C for 2 min was used for AmpErase incubation followed by 15 min at 95°C to inactivate AmpErase and to activate the AmpliTaq. Cycling conditions were as follows: melting step at 95°C for 1 min, annealing at 58°C for 1 min and elongation at 72°C, for 40 cycles. At the end of the PCR run, a dissociation curve was determined by ramping the temperature of the sample from 60 to 95°C while continuously collecting fluorescence data. Non template controls were included for each primer pair to check for any significant levels of contaminants. Values were normalized by the mean of the 3 reference genes hypoxanthine-guanine phosphoribosyltransferase, elongation factor 1-a and ribosomal protein S27a.

### Statistical analysis

Normality of the distribution was tested by Kolmogorov-Smirnov test. Statistical significance was calculated by using *t-*test of 3–5 independent retinas (20 sections)/genotype/age. Values are expressed as mean ± s.e.m. Values of *P<0.05, **P<0.01, ***P<0.001 were considered to be statistically significant. Calculations and graphs were generated using GraphPad Prism 5.

## Supporting Information

Figure S1 Retinal activity in *Crb1Crb2* mutant retinas is severely impaired. (A–C) Single-flash electroretinogram age series in double heterozygote *Crb1^+/−^Crb2^F/+^* cKO (blue), *Crb1^+/−^Crb2* cKO (purple) and *Crb1Crb2* cKO mice (red) at 1M (A), 3M (B) and 6M (C). Scotopic b-wave amplitudes were plotted as a function of the logarithm of the flash intensity. Boxes indicate the 25% and 75% quantile range, whiskers indicate the 5% and 95% quantiles, and solid lines connect the medians of the data. In affected *Crb1^+/−^Crb2* cKO and *Crb1Crb2* cKO mice, the b-wave amplitude was already considerably reduced at the age of 1M under both scotopic and photopic conditions, and declined even at 3M and 6M to a flat electroretinogram.
(TIF)

Figure S2 *In vivo* retinal imaging in *Crb1^+/−^Crb2* cKO mice. *Crb1^+/−^Crb2* cKO mice were examined with scanning laser ophthalmoscopy (A,D,G), spectral domain optical coherence tomography (B,C,E,F,H,I) at the age of 1M (A–C). 3M (D–F) and 6M (G–I). Due to the fact that younger mice from this genotype did not show morphological alterations (data not shown), 12M *Crb1^+/−^Crb2^F/+^* cKO were used as controls, and even here no abnormalities were found neither in the native fundus image, nor in the autofluorescence or the retinal vasculature (Figure 3). The retinal organization was also unaffected, as observed by optical coherence tomography analysis (Figure 3). *Crb1^+/−^Crb2* cKO animals already at 1M showed a spotty fundus, as well as several degeneration sites represented by the presence of fluorescent material detectable at 488 nm (A). In the optical coherence tomography analysis, a decrease in the retinal thickness was observed as well as a wavy appearance of the outer plexiform layer together with the formation of structures like rosettes located in the outer nuclear layer (B,C). At 3M, the retinal thickness was further decreased, specially at the level of the outer nuclear layer (E,F). In the autofluorescence image, many hyper and hypo fluorescent regions as well as a several vascular changes indicating neovascularization processes were observed (D). Six month old individuals presented a more severe degeneration ascertained by scanning laser ophthalmoscopy (G) and optical coherence tomography (H,I). Abbreviations: AF, autofluorescence; d, dorsal; FA, fluorescein angiography; RF, red free; v, ventral.
(TIF)

Figure S3 Loss of Crumbs complex and adherens junctions, ectopic synapses and cell death in *Crb1Crb2* cKO retina. Confocal immunohistofluorescent representative pictures of CRB1 and CRB2, adherens junction marker (Nectin1), Crumbs complex members (PALS1 and MUPP1), OPL ribbon synapse markers (PSD95 and PKCα for bipolar cells) in control (left panel) and *Crb1Crb2* cKO (right panel) retinas at P14 (A–D). Adherens junctions and CRB complex proteins were totally absent in the subapical region, except in photoreceptor rosettes which contained few wild type cells still expressing CRB2 in *Crb1Crb2* cKO (A–B, D; white arrowheads). The synapses between photoreceptor and bipolar cells located normally in the OPL were found ectopically localized throughout the retina thickness in *Crb1Crb2* cKO (C; white arrowheads). Confocal immunohistofluorescent representative pictures of apoptotic cells (cCaspase 3) in the nuclear layer of *Crb1Crb2* cKO at P14 (E) and 3M (F). Cleaved caspase 3 positive cells were rods (Rhodopsin) at P14 and mainly bipolar cells (Chx10*Cre-GFP*) at 3M. GCL, ganglion cell layer; INL, inner nuclear layer; NL, nuclear layer; ONL, outer nuclear layer; OPL, outer plexiform layer; RPE, retinal pigmented epithelium; SAR, subapical region. Scale bar: 50 µm (A–D); 25 µm (E–F).
(TIF)

Figure S4 Loss of adherens junctions, CRB and PAR complexes in embryonic *Crb1Crb2* cKO retina. Confocal immunohistofluorescent representative pictures of CRB2 (D), adherens junction marker (Nectin1, B), CRB complex member (PALS1, A) and PAR complex member (PAR3, C) of control (left panel) and *Crb1Crb2* cKO (right panel) retinas at E15.5. Areas with completely disrupted outer limiting membrane showed loss of expression of adherens junction, CRB and PAR complex markers, except in pseudo-rosettes of progenitor cells which contained few wild type cells still expressing CRB2. Electron microscopic zoom pictures at the adherens junctions of E17.5 littermate control (E) and *Crb1Crb2* cKO (F) retinas. *Crb1Crb2* cKO retinas showed completely absence of adherens junctions at the outer limiting membrane. GCL, ganglion cell layer; NBL, neuroblast layer; RPE, retinal pigmented epithelium; SAR, subapical region. Scale bar: 50 µm (A–D); 1 µm (E–F).
(TIF)

Figure S5 Ectopic localization of cell types in *Crb1^+/−^Crb2* cKO and *Crb1Crb2* cKO retinas. The cell types were immunostained with Brn3b for ganglion cells (A), cone arrestin (CAR) for cone photoreceptors (B), choline acetyltransferase for early born cholinergic amacrine cells (C), Sox9 and glutamine synthetase for Müller cells (E) and PKCα and nuclear *Cre-GFP* under the Chx10 promoter for bipolar cells (F) at P14 and Rhodopsin for rod photoreceptors at P10 (D) in control and *Crb1Crb2* cKO. Some ectopic ganglion and cholinergic-amacrine cells localize in rosettes in the vicinity of the retinal pigment epithelium and established dendrites in the lumen. Few ectopic cone photoreceptors are found in the ganglion cell layer. In contrast, the late born rod photoreceptors, Müller glial cells and bipolar cells localize in the two thick nuclear layers. Retinal sections are stained with rhodopsin for rods and cone arrestin for cones and the presence of nuclear GFP for bipolar cells is due to the *Cre-GFP* under the Chx10 promoter in the Chx10*Cre* transgenic line in *Crb1^+/−^Crb2* cKO retinas at P10 (G–H). Rod and cone photoreceptors are present in the rosettes and segments are present in the lumen. The cells that ectopically localize in the ganglion cell layer in these mutant mice are rod and cone photoreceptors and bipolar cells (H). Retinal sections are stained with calretinin for ganglion and amacrine cells and bipolar cells with the nuclear Cre- GFP in *Crb1^+/−^Crb2* cKO retinas at P10 (I–J). The second type of rosettes is formed of ganglion and amacrine cells surrounded by bipolar cells. GCL, ganglion cell layer; INL, inner nuclear layer; NL, nuclear layer; ONL, outer nuclear layer. Scale bar: 50 µm.
(TIF)

Figure S6 Overproliferation of retinal progenitor cells in *Crb1^+/−^Crb2* cKO and *Crb1Crb2* cKO retinas. The number of mitotic cells immunostained with anti-phospho-Histone H3 (pH3; A) and apoptotic cells immunostained with cleaved caspase 3 (B) were quantified from E15.5 to P5 in 10–15 representative pictures of whole retinas from 3–5 control and *Crb1^+/−^Crb2* cKO retinas. Mutant retinas showed a significant increase in the number of mitotic and apoptotic cells at E17.5. At P5 an increase in the number of mitotic and a decrease in the number of apoptotic cells are observed. Mitotic cells were immunostained with pH3 immunostaining (M-phase) and Ki67 immunostaining (M, G2, S and late G1 phases) and counterstained with DAPI in representative pictures of control (left panel) and *Crb1Crb2* cKO (right panel) retinas at E17.5 (C) and in the center of the retina at P5 (D). The mitotic cells displayed an aberrant distribution in *Crb1Crb2* cKO retinas, especially the M-phase cells in E17.5 retina, which in the control localized at the outer limiting membrane whereas in *Crb1Crb2* cKO retina these cells had a scattered distribution throughout the whole neuroblast layer (C). At P5 in the centre of the retina few Ki67^+^ cells were detected, whereas in *Crb1Crb2* cKO retina many cells were still dividing especially in M-phase (D). Immunostaining of E17.5 retinal sections of control and *Crb1Crb2* cKO was performed with Ki67 and BrdU antibodies after 30 min pulse (E) or 24 hours (F) of BrdU labelling and counterstained with DAPI for nuclear staining. Ki67 and BrdU positive cells localized through the entire thickness of *Crb1Crb2* cKO. Flow cytometry profiles of control and *Crb1Crb2* cKO retinal cells at E17.5 (G) based on Ki67 labelling and DNA content. The number of total proliferating cells using Ki67 immunostaining is increased by a factor two in *Crb1Crb2* cKO retinas at P5 (H). Scale bar: 50 µm. ***P<0.001.
(TIF)

Figure S7 CRB1 and CRB2 act on proliferative signalling pathways. Representative pictures of P120-catenin (A), β-catenin (B) and YAP with *Chx10Cre-GFP* (C) of control and *Crb1Crb2* cKO retinas at E17.5. P120- and β-catenins, which normally localized at the adherens junctions were found in ectopic rosette structures (white arrowheads). YAP protein normally localized at the subapical region adjacent to adherens junctions and in the nuclei of the retinal progenitors (colocalization with Chx10*Cre-GFP*) whereas YAP was found only in ectopic rosette structures (white arrowheads) similarly to the catenins and in the cytoplasm of the retinal progenitors in *Crb1Crb2* cKO retinas. Quantification of the number of phospho-ribosomal protein positive cells (D) showed no difference between control and *Crb1Crb2* cKO retinas at E17.5 and P1. Representative Western Blot of YAP, pYAP, Notch intracellular domain (NICD), Kaiso, β-catenin (β-catn), P120-catenin (P120-catn), actin and Glyceraldehyde 3-phosphate dehydrogenase (GAPDH) at E17.5 (E) and P1 (F). Scale bar: 50 µm. Values are presented as mean ± s.e.m.
(TIF)

Table S1 Antibody list and dilution used for immunohistochemistry.
(DOC)

Text S1 Detailed Materials and Methods of *In vivo* analysis, immunohistochemical analysis and flow cytometry.
(DOC)

## References

[1] Andreazzoli M (2009) Molecular regulation of vertebrate retina cell fate. Birth Defects Res C Embryo Today 87: 284–295. 19750521 10.1002/bdrc.20161

[2] Livesey FJ, Cepko CL (2001) Vertebrate neural cell-fate determination: lessons from the retina. Nat Rev Neurosci 2: 109–118. 11252990 10.1038/35053522

[3] Young RW (1985) Cell differentiation in the retina of the mouse. Anat Rec 212: 199–205. 3842042 10.1002/ar.1092120215

[4] Agathocleous M, Harris WA (2009) From progenitors to differentiated cells in the vertebrate retina. Annu Rev Cell Dev Biol 25: 45–69. 19575661 10.1146/annurev.cellbio.042308.113259

[5] Burmeister M, Novak J, Liang MY, Basu S, Ploder L, et al (1996) Ocular retardation mouse caused by Chx10 homeobox null allele: impaired retinal progenitor proliferation and bipolar cell differentiation. Nat Genet 12: 376–384. 8630490 10.1038/ng0496-376

[6] Martin-Belmonte F, Perez-Moreno M (2011) Epithelial cell polarity, stem cells and cancer. Nat Rev Cancer 12: 23–38. 22169974 10.1038/nrc3169

[7] Tepass U, Theres C, Knust E (1990) crumbs encodes an EGF-like protein expressed on apical membranes of Drosophila epithelial cells and required for organization of epithelia. Cell 61: 787–799. 2344615 10.1016/0092-8674(90)90189-l

[8] Bulgakova NA, Knust E (1990) The Crumbs complex: from epithelial-cell polarity to retinal degeneration. J Cell Sci 122: 2587–2596. 19625503 10.1242/jcs.023648

[9] Kim S, Lehtinen MK, Sessa A, Zappaterra MW, Cho SH, et al (2010) The apical complex couples cell fate and cell survival to cerebral cortical development. Neuron 66: 69–84. 20399730 10.1016/j.neuron.2010.03.019 PMC2872122

[10] Herranz H, Stamataki E, Feiguin F, Milán M (2006) Self-refinement of Notch activity through the transmembrane protein Crumbs: modulation of gamma-secretase activity. EMBO Rep 7: 297–302. 16440003 10.1038/sj.embor.7400617 PMC1456882

[11] Mitsuishi Y, Hasegawa H, Matsuo A, Araki W, Suzuki T, et al (2010) Human CRB2 inhibits gamma-secretase cleavage of amyloid precursor protein by binding to the presenilin complex. J Biol Chem 285: 14920–14931. 20299451 10.1074/jbc.M109.038760 PMC2865292

[12] Ohata S, Aoki R, Kinoshita S, Yamaguchi M, Tsuruoka-Kinoshita S, et al (2011) Dual roles of Notch in regulation of apically restricted mitosis and apicobasal polarity of neuroepithelial cells. Neuron 69: 215–230. 21262462 10.1016/j.neuron.2010.12.026

[13] Massey-Harroche D, Delgrossi MH, Lane-Guermonprez L, Arsanto JP, Borg JP, et al (2007) Evidence for a molecular link between the tuberous sclerosis complex and the Crumbs complex. Hum Mol Genet 16: 529–536. 17234746 10.1093/hmg/ddl485

[14] Zhao B, Tumaneng K, Guan KL (2011) The Hippo pathway in organ size control, tissue regeneration and stem cell self-renewal. Nat Cell Biol 13: 877–883. 21808241 10.1038/ncb2303 PMC3987945

[15] Chen CL, Gajewski KM, Hamaratoglu F, Bossuyt W, Sansores-Garcia L, et al (2010) The apical-basal cell polarity determinant Crumbs regulates Hippo signaling in Drosophila. Proc Natl Acad Sci U S A 107: 15810–15815. 20798049 10.1073/pnas.1004060107 PMC2936591

[16] Robinson BS, Huang J, Hong Y, Moberg KH (2010) Crumbs regulates Salvador/Warts/Hippo signaling in Drosophila via the FERM-domain protein Expanded. Curr Biol 20: 582–590. 20362445 10.1016/j.cub.2010.03.019 PMC2855393

[17] Varelas X, Samavarchi-Tehrani P, Narimatsu M, Weiss A, Cockburn K, et al (2010) The Crumbs complex couples cell density sensing to Hippo-dependent control of the TGF-β-SMAD pathway. Dev Cell 19: 831–844. 21145499 10.1016/j.devcel.2010.11.012

[18] Richard M, Roepman R, Aartsen WM, van Rossum AG, den Hollander AI, et al (2006) Towards understanding CRUMBS function in retinal dystrophies. Hum Mol Genet 15: 235–243. 10.1093/hmg/ddl195 16987889

[19] den Hollander AI, Roepman R, Koenekoop RK, Cremers FP (2008) Leber congenital amaurosis: genes, proteins and disease mechanisms. Prog Retin Eye Res 27: 391–419. 18632300 10.1016/j.preteyeres.2008.05.003

[20] Jacobson SG, Cideciyan AV, Aleman TS, Pianta MJ, Sumaroka A, et al (2003) Crumbs homolog 1 (*CRB1*) mutations result in a thick human retina with abnormal lamination. Hum Mol Genet 12: 1073–1078. 12700176 10.1093/hmg/ddg117

[21] den Hollander AI, Ghiani M, de Kok YJ, Wijnholds J, Ballabio A, et al (2002) Isolation of *Crb1*, a mouse homologue of Drosophila crumbs, and analysis of its expression pattern in eye and brain. Mech Dev 110: 203–207. 11744384 10.1016/s0925-4773(01)00568-8

[22] Alves CH, Sanz Sanz A, Park B, Pellissier LP, Tanimoto N, et al (2013) Loss of CRB2 in the mouse retina mimics human Retinitis Pigmentosa due to mutations in the *CRB1* gene. Hum Mol Genet 22: 35–50. 23001562 10.1093/hmg/dds398

[23] van Rossum AG, Aartsen WM, Meuleman J, Klooster J, Malysheva A, et al (2006) Pals1/Mpp5 is required for correct localization of *Crb1* at the subapical region in polarized Muller glia cells. Hum Mol Genet 15: 2659–2672. 16885194 10.1093/hmg/ddl194

[24] van de Pavert SA, Kantardzhieva A, Malysheva A, Meuleman J, Versteeg I, et al (2004) Crumbs homologue 1 is required for maintenance of photoreceptor cell polarization and adhesion during light exposure. J Cell Sci 117: 4169–4177. 15316081 10.1242/jcs.01301

[25] van de Pavert SA, Sanz AS, Aartsen WM, Vos RM, Versteeg I, et al (2007) *Crb1* is a determinant of retinal apical Müller glia cell features. Glia 55: 1486–1497. 17705196 10.1002/glia.20561

[26] van de Pavert SA, Meuleman J, Malysheva A, Aartsen WM, Versteeg I, et al (2007) A single amino acid substitution (Cys249Trp) in *Crb1* causes retinal degeneration and deregulates expression of pituitary tumor transforming gene Pttg1. J Neurosci 27: 564–573. 17234588 10.1523/JNEUROSCI.3496-06.2007 PMC6672796

[27] Mehalow AK, Kameya S, Smith RS, Hawes NL, Denegre JM, et al (2003) *CRB1* is essential for external limiting membrane integrity and photoreceptor morphogenesis in the mammalian retina. Hum Mol Genet 12: 2179–2189. 12915475 10.1093/hmg/ddg232

[28] Rowan S, Cepko CL (2004) Genetic analysis of the homeodomain transcription factor Chx10 in the retina using a novel multifunctional BAC transgenic mouse reporter. Dev Biol 271: 388–402. 15223342 10.1016/j.ydbio.2004.03.039

[29] Sakagami K, Gan L, Yang XJ (2009) Distinct effects of Hedgehog signaling on neuronal fate specification and cell cycle progression in the embryonic mouse retina. J Neurosci 29: 6932–6944. 19474320 10.1523/JNEUROSCI.0289-09.2009 PMC2715855

[30] Das G, Choi Y, Sicinski P, Levine EM (2009) Cyclin D1 fine-tunes the neurogenic output of embryonic retinal progenitor cells. Neural Dev 4: 15. 19416500 10.1186/1749-8104-4-15 PMC2694796

[31] Park JI, Kim SW, Lyons JP, Ji H, Nguyen TT, et al (2005) Kaiso/p120-catenin and TCF/beta-catenin complexes coordinately regulate canonical Wnt gene targets. Dev Cell 8: 843–854. 15935774 10.1016/j.devcel.2005.04.010

[32] Park JI, Ji H, Jun S, Gu D, Hikasa H, et al (2006) Frodo links Dishevelled to the p120-catenin/Kaiso pathway: distinct catenin subfamilies promote Wnt signals. Dev Cell 11: 683–695. 17084360 10.1016/j.devcel.2006.09.022

[33] Zhang H, Deo M, Thompson RC, Uhler MD, Turner DL (2012) Negative regulation of Yap during neuronal differentiation. Dev Biol 361: 103–115. 22037235 10.1016/j.ydbio.2011.10.017 PMC3235039

[34] Reese BE (2011) Development of the retina and optic pathway. Vision Res 51: 613–632. 20647017 10.1016/j.visres.2010.07.010 PMC2974959

[35] Cho SH, Kim JY, Simons DL, Song JY, Le JH, et al (2012) Genetic ablation of Pals1 in retinal progenitor cells models the retinal pathology of Leber congenital amaurosis. Hum Mol Genet 21: 2663–2676. 22398208 10.1093/hmg/dds091 PMC3363335

[36] Park B, Alves CH, Lundvig DM, Tanimoto N, Beck SC, et al (2011) PALS1 is essential for retinal pigment epithelium structure and neural retina stratification. J Neurosci 31: 17230–17241. 22114289 10.1523/JNEUROSCI.4430-11.2011 PMC6623860

[37] Chartier NT, Oddou CI, Lainé MG, Ducarouge B, Marie CA, et al (2007) Cyclin-dependent kinase 2/cyclin E complex is involved in p120 catenin (p120ctn)-dependent cell growth control: a new role for p120ctn in cancer. Cancer Res 67: 9781–9790. 17942908 10.1158/0008-5472.CAN-07-0233 PMC2695941

[38] Jiang G, Wang Y, Dai S, Liu Y, Stoecker M, et al (2012) P120-catenin isoforms 1 and 3 regulate proliferation and cell cycle of lung cancer cells via β-catenin and Kaiso respectively. PLoS One 7: e30303. 22276175 10.1371/journal.pone.0030303 PMC3262806

[39] Aleman TS, Cideciyan AV, Aguirre GK, Huang WC, Mullins CL, et al (2011) Human *CRB1*-associated retinal degeneration: comparison with the rd8 *Crb1*-mutant mouse model. Invest Ophthalmol Vis Sci 52: 6898–6910. 21757580 10.1167/iovs.11-7701 PMC3176016

[40] Trifunovic D, Karali M, Camposampiero D, Ponzin D, Banfi S, et al (2008) A high-resolution RNA expression atlas of retinitis pigmentosa genes in human and mouse retinas. Invest Ophthalmol Vis Sci 49: 2330–2336. 18281612 10.1167/iovs.07-1513

[41] Bibb LC, Holt JK, Tarttelin EE, Hodges MD, Gregory-Evans K, et al (2001) Temporal and spatial expression patterns of the CRX transcription factor and its downstream targets. Critical differences during human and mouse eye development. Hum Mol Genet 10: 1571–1579. 11468275 10.1093/hmg/10.15.1571

[42] Donovan SL, Schweers B, Martins R, Johnson D, Dyer MA (2006) Compensation by tumor suppressor genes during retinal development in mice and humans. BMC Biol 4: 14. 16672052 10.1186/1741-7007-4-14 PMC1481602

[43] Bujakowska K, Audo I, Mohand-Saïd S, Lancelot ME, Antonio A, et al (2012) *CRB1* mutations in inherited retinal dystrophies. Hum Mutat 33: 306–315. 22065545 10.1002/humu.21653 PMC3293109

[44] van den Hurk JA, Rashbass P, Roepman R, Davis J, Voesenek KE, et al (2005) Characterization of the Crumbs homolog 2 (CRB2) gene and analysis of its role in retinitis pigmentosa and Leber congenital amaurosis. Mol Vis 11: 263–273. 15851977

[45] Tanimoto N, Muehlfriedel RL, Fischer MD, Fahl E, Humphries P, et al (2009) Vision tests in the mouse: functional phenotyping with electroretinography. Front Biosci 14: 2730–2737. 10.2741/3409 19273231

[46] Seeliger MW, Beck SC, Pereyra-Muñoz N, Dangel S, Tsai JY, et al (2005) In vivo confocal imaging of the retina in animal models using scanning laser ophthalmoscopy. Vision Res 45: 3512–3519. 16188288 10.1016/j.visres.2005.08.014

[47] Fischer MD, Huber G, Beck SC, Tanimoto N, Muehlfriedel R, et al (2009) Non invasive, in vivo assessment of mouse retinal structure using optical coherence tomography. PLoS One 4: e7507. 19838301 10.1371/journal.pone.0007507 PMC2759518

