# Supplementary figures and images for: Targeted Ablation of Crb1 and Crb2 in Retinal Progenitor Cells Mimics Leber Congenital Amaurosis
Source: PLoS Genet. 2013 Dec 5;9(12):e1003976. doi: 10.1371/journal.pgen.1003976 (PMC3854796; doi:10.1371/journal.pgen.1003976)

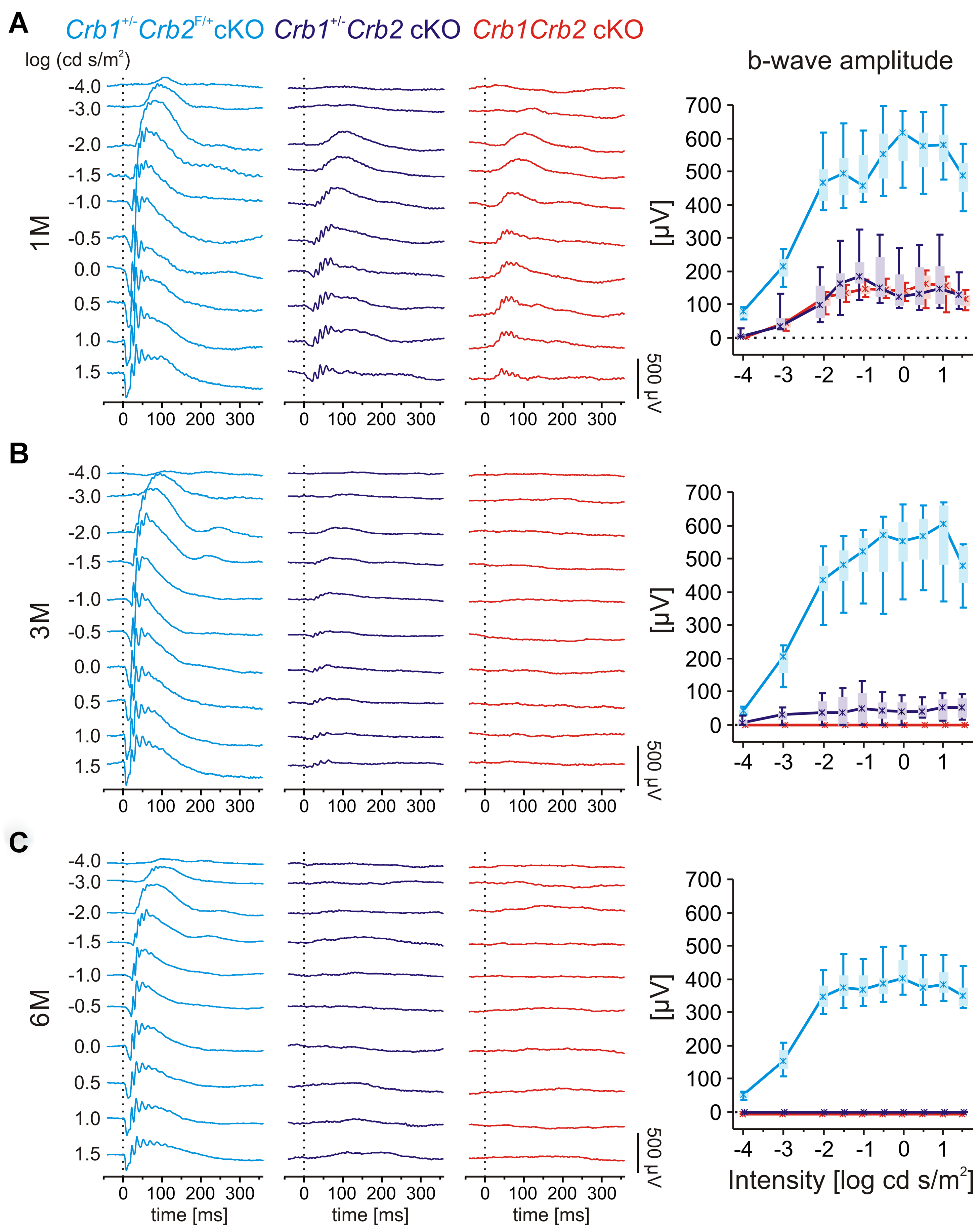

Supplement: Figure S1 — Retinal activity in Crb1Crb2 mutant retinas is severely impaired. (A–C) Single-flash electroretinogram age series in double heterozygote Crb1+/−Crb2F/+ cKO (blue), Crb1+/−Crb2 cKO (purple) and Crb1Crb2 cKO mice (red) at 1M (A), 3M (B) and 6M (C). Scotopic b-wave amplitudes were plotted as a function of the logarithm of the flash intensity. Boxes indicate the 25% and 75% quantile range, whiskers indicate the 5% and 95% quantiles, and solid lines connect the medians of the data. In affected Crb1+/−Crb2 cKO and Crb1Crb2 cKO mice, the b-wave amplitude was already considerably reduced at the age of 1M under both scotopic and photopic conditions, and declined even at 3M and 6M to a flat electroretinogram. (TIF) [file pgen.1003976.s001.tif]

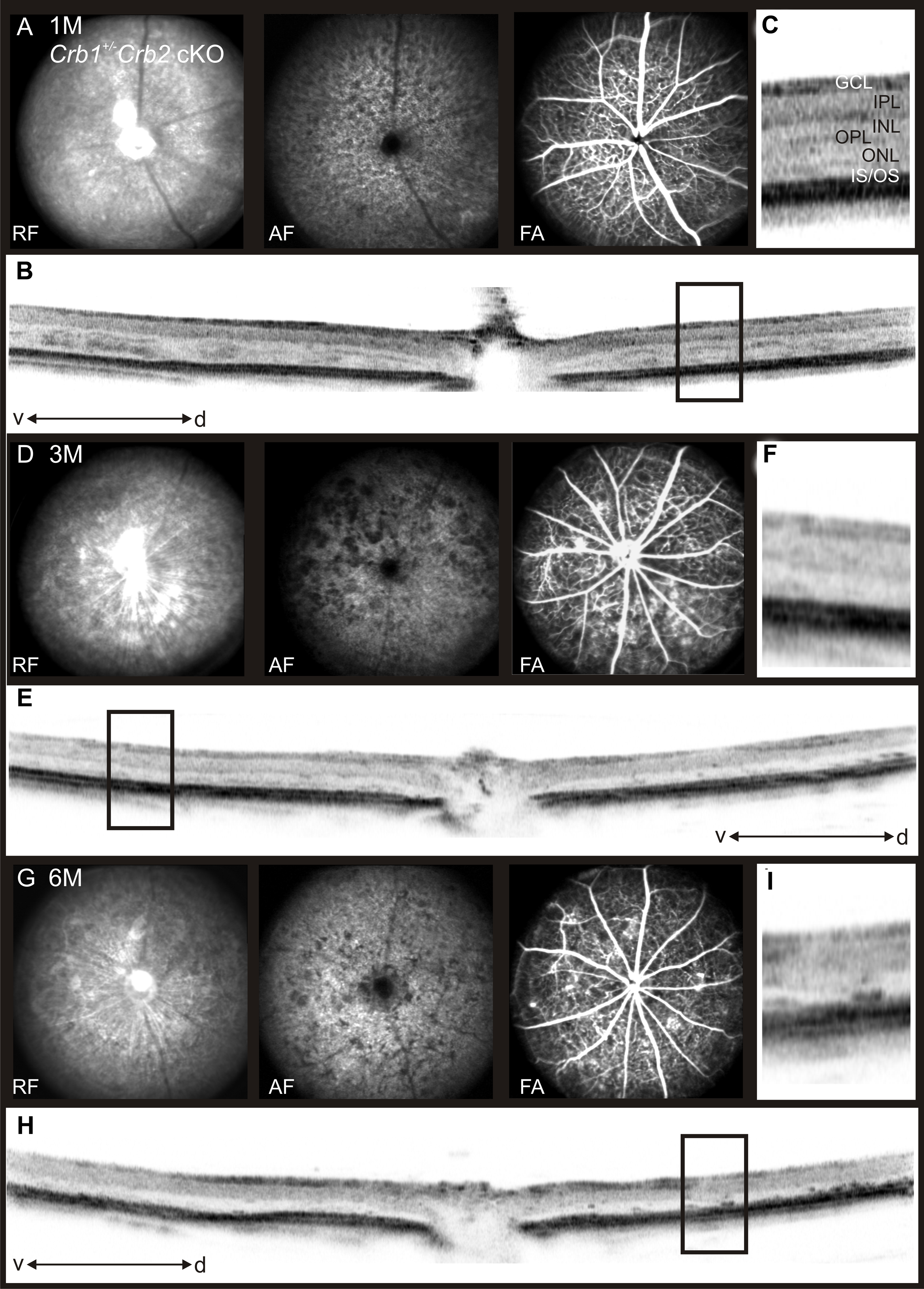

Supplement: Figure S2 — In vivo retinal imaging in Crb1+/−Crb2 cKO mice. Crb1+/−Crb2 cKO mice were examined with scanning laser ophthalmoscopy (A,D,G), spectral domain optical coherence tomography (B,C,E,F,H,I) at the age of 1M (A–C). 3M (D–F) and 6M (G–I). Due to the fact that younger mice from this genotype did not show morphological alterations (data not shown), 12M Crb1+/−Crb2F/+ cKO were used as controls, and even here no abnormalities were found neither in the native fundus image, nor in the autofluorescence or the retinal vasculature (Figure 3). The retinal organization was also unaffected, as observed by optical coherence tomography analysis (Figure 3). Crb1+/−Crb2 cKO animals already at 1M showed a spotty fundus, as well as several degeneration sites represented by the presence of fluorescent material detectable at 488 nm (A). In the optical coherence tomography analysis, a decrease in the retinal thickness was observed as well as a wavy appearance of the outer plexiform layer together with the formation of structures like rosettes located in the outer nuclear layer (B,C). At 3M, the retinal thickness was further decreased, specially at the level of the outer nuclear layer (E,F). In the autofluorescence image, many hyper and hypo fluorescent regions as well as a several vascular changes indicating neovascularization processes were observed (D). Six month old individuals presented a more severe degeneration ascertained by scanning laser ophthalmoscopy (G) and optical coherence tomography (H,I). Abbreviations: AF, autofluorescence; d, dorsal; FA, fluorescein angiography; RF, red free; v, ventral. (TIF) [file pgen.1003976.s002.tif]

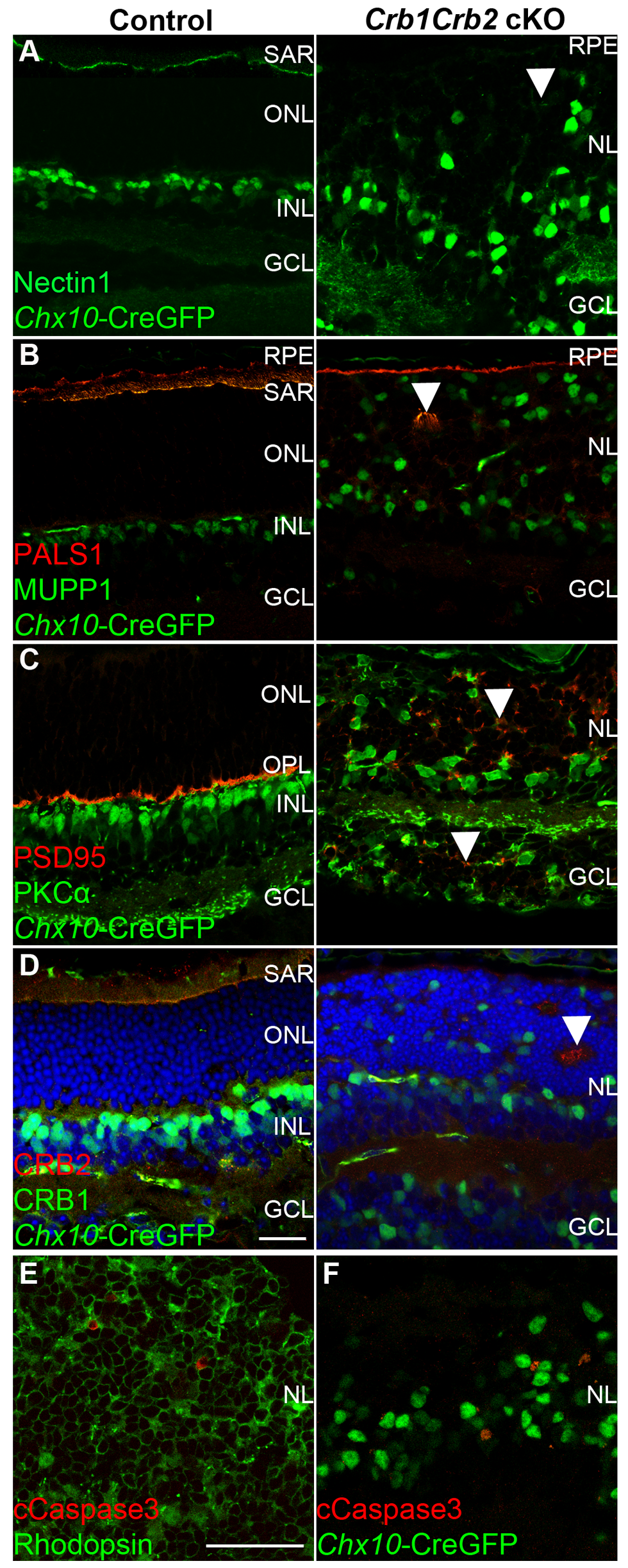

Supplement: Figure S3 — Loss of Crumbs complex and adherens junctions, ectopic synapses and cell death in Crb1Crb2 cKO retina. Confocal immunohistofluorescent representative pictures of CRB1 and CRB2, adherens junction marker (Nectin1), Crumbs complex members (PALS1 and MUPP1), OPL ribbon synapse markers (PSD95 and PKCα for bipolar cells) in control (left panel) and Crb1Crb2 cKO (right panel) retinas at P14 (A–D). Adherens junctions and CRB complex proteins were totally absent in the subapical region, except in photoreceptor rosettes which contained few wild type cells still expressing CRB2 in Crb1Crb2 cKO (A–B, D; white arrowheads). The synapses between photoreceptor and bipolar cells located normally in the OPL were found ectopically localized throughout the retina thickness in Crb1Crb2 cKO (C; white arrowheads). Confocal immunohistofluorescent representative pictures of apoptotic cells (cCaspase 3) in the nuclear layer of Crb1Crb2 cKO at P14 (E) and 3M (F). Cleaved caspase 3 positive cells were rods (Rhodopsin) at P14 and mainly bipolar cells (Chx10Cre-GFP) at 3M. GCL, ganglion cell layer; INL, inner nuclear layer; NL, nuclear layer; ONL, outer nuclear layer; OPL, outer plexiform layer; RPE, retinal pigmented epithelium; SAR, subapical region. Scale bar: 50 µm (A–D); 25 µm (E–F). (TIF) [file pgen.1003976.s003.tif]

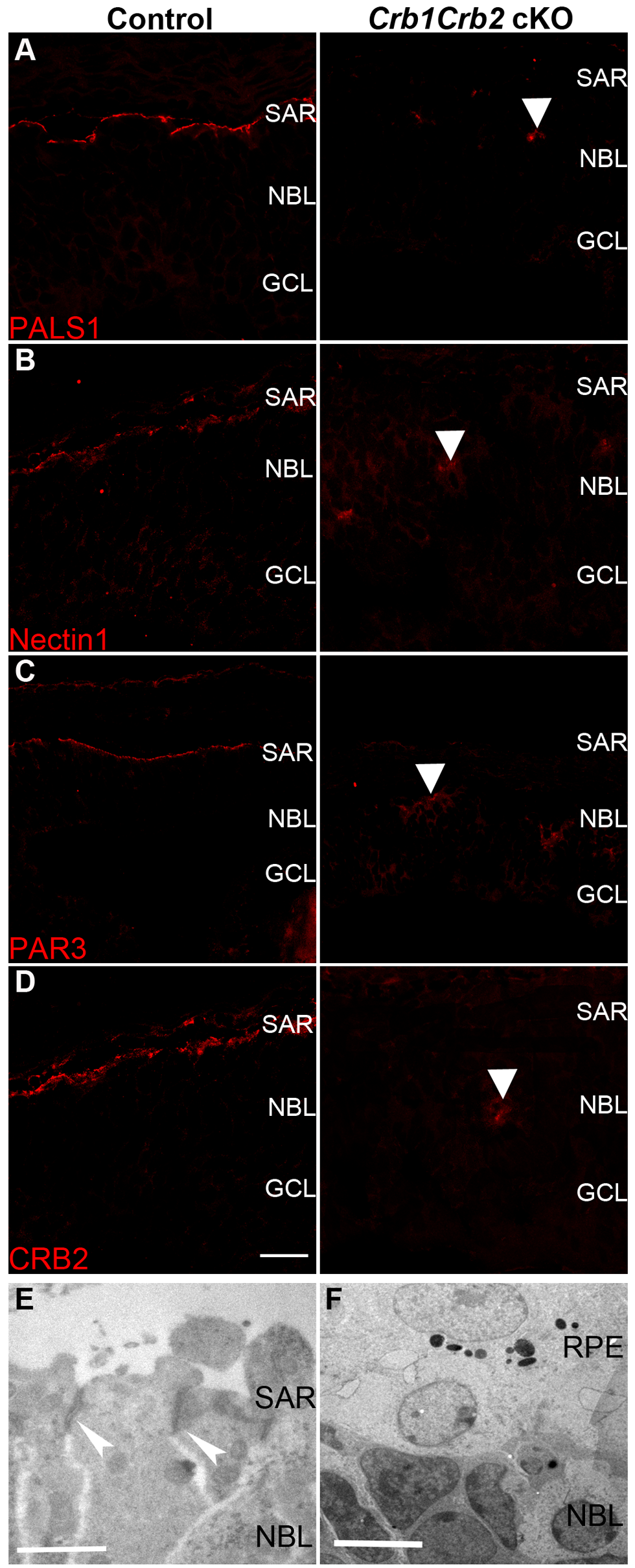

Supplement: Figure S4 — Loss of adherens junctions, CRB and PAR complexes in embryonic Crb1Crb2 cKO retina. Confocal immunohistofluorescent representative pictures of CRB2 (D), adherens junction marker (Nectin1, B), CRB complex member (PALS1, A) and PAR complex member (PAR3, C) of control (left panel) and Crb1Crb2 cKO (right panel) retinas at E15.5. Areas with completely disrupted outer limiting membrane showed loss of expression of adherens junction, CRB and PAR complex markers, except in pseudo-rosettes of progenitor cells which contained few wild type cells still expressing CRB2. Electron microscopic zoom pictures at the adherens junctions of E17.5 littermate control (E) and Crb1Crb2 cKO (F) retinas. Crb1Crb2 cKO retinas showed completely absence of adherens junctions at the outer limiting membrane. GCL, ganglion cell layer; NBL, neuroblast layer; RPE, retinal pigmented epithelium; SAR, subapical region. Scale bar: 50 µm (A–D); 1 µm (E–F). (TIF) [file pgen.1003976.s004.tif]

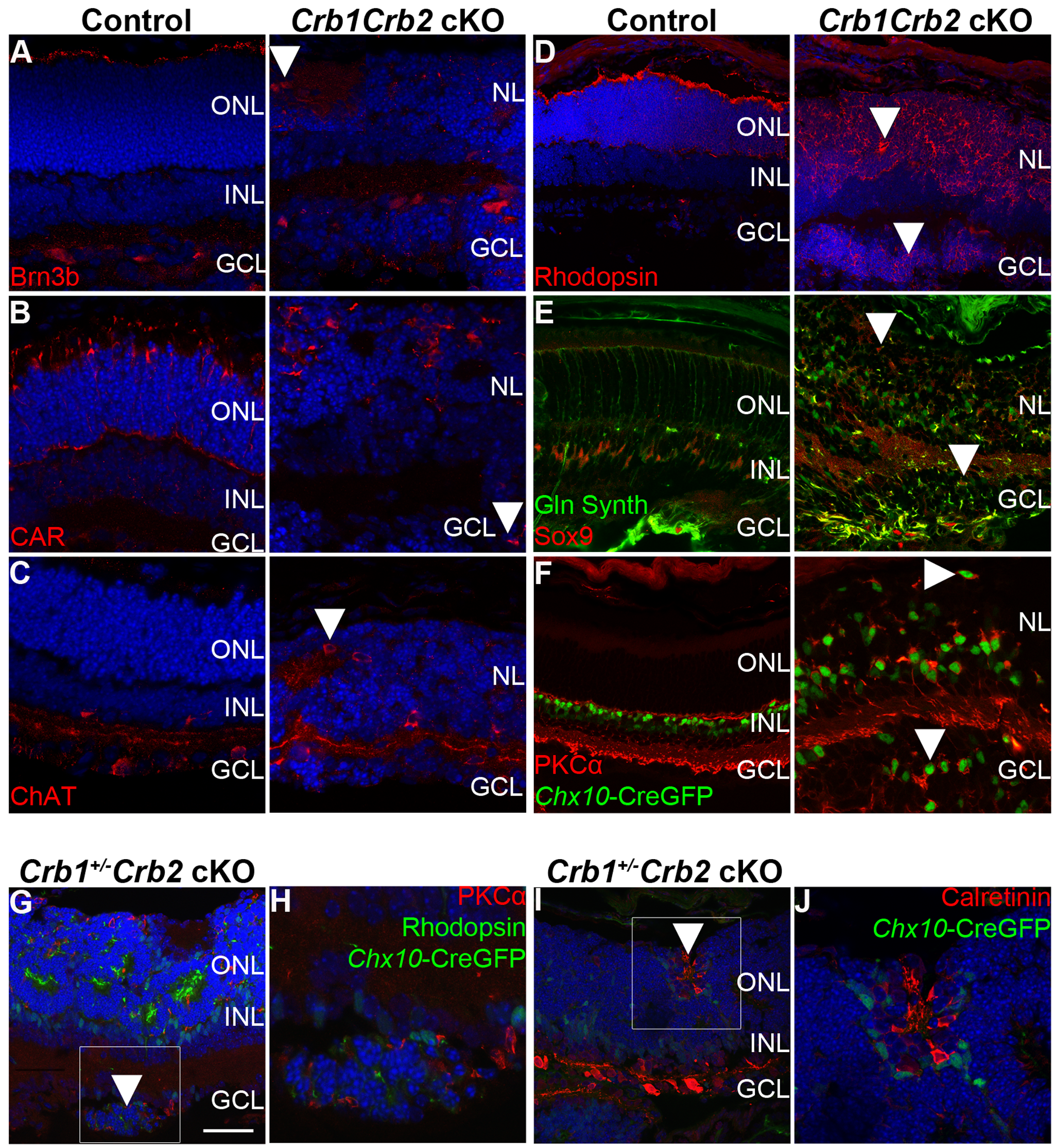

Supplement: Figure S5 — Ectopic localization of cell types in Crb1+/−Crb2 cKO and Crb1Crb2 cKO retinas. The cell types were immunostained with Brn3b for ganglion cells (A), cone arrestin (CAR) for cone photoreceptors (B), choline acetyltransferase for early born cholinergic amacrine cells (C), Sox9 and glutamine synthetase for Müller cells (E) and PKCα and nuclear Cre-GFP under the Chx10 promoter for bipolar cells (F) at P14 and Rhodopsin for rod photoreceptors at P10 (D) in control and Crb1Crb2 cKO. Some ectopic ganglion and cholinergic-amacrine cells localize in rosettes in the vicinity of the retinal pigment epithelium and established dendrites in the lumen. Few ectopic cone photoreceptors are found in the ganglion cell layer. In contrast, the late born rod photoreceptors, Müller glial cells and bipolar cells localize in the two thick nuclear layers. Retinal sections are stained with rhodopsin for rods and cone arrestin for cones and the presence of nuclear GFP for bipolar cells is due to the Cre-GFP under the Chx10 promoter in the Chx10Cre transgenic line in Crb1+/−Crb2 cKO retinas at P10 (G–H). Rod and cone photoreceptors are present in the rosettes and segments are present in the lumen. The cells that ectopically localize in the ganglion cell layer in these mutant mice are rod and cone photoreceptors and bipolar cells (H). Retinal sections are stained with calretinin for ganglion and amacrine cells and bipolar cells with the nuclear Cre- GFP in Crb1+/−Crb2 cKO retinas at P10 (I–J). The second type of rosettes is formed of ganglion and amacrine cells surrounded by bipolar cells. GCL, ganglion cell layer; INL, inner nuclear layer; NL, nuclear layer; ONL, outer nuclear layer. Scale bar: 50 µm. (TIF) [file pgen.1003976.s005.tif]

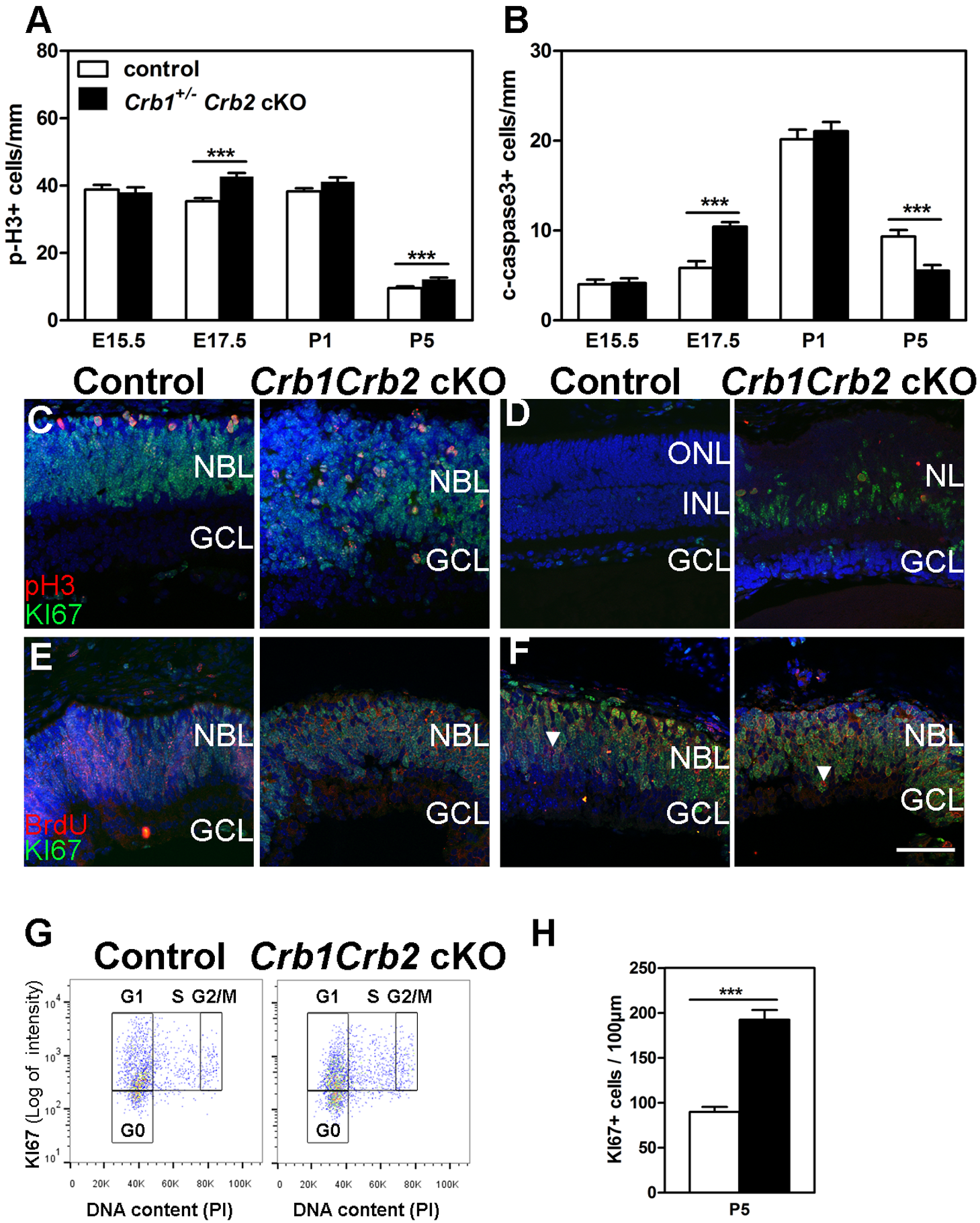

Supplement: Figure S6 — Overproliferation of retinal progenitor cells in Crb1+/−Crb2 cKO and Crb1Crb2 cKO retinas. The number of mitotic cells immunostained with anti-phospho-Histone H3 (pH3; A) and apoptotic cells immunostained with cleaved caspase 3 (B) were quantified from E15.5 to P5 in 10–15 representative pictures of whole retinas from 3–5 control and Crb1+/−Crb2 cKO retinas. Mutant retinas showed a significant increase in the number of mitotic and apoptotic cells at E17.5. At P5 an increase in the number of mitotic and a decrease in the number of apoptotic cells are observed. Mitotic cells were immunostained with pH3 immunostaining (M-phase) and Ki67 immunostaining (M, G2, S and late G1 phases) and counterstained with DAPI in representative pictures of control (left panel) and Crb1Crb2 cKO (right panel) retinas at E17.5 (C) and in the center of the retina at P5 (D). The mitotic cells displayed an aberrant distribution in Crb1Crb2 cKO retinas, especially the M-phase cells in E17.5 retina, which in the control localized at the outer limiting membrane whereas in Crb1Crb2 cKO retina these cells had a scattered distribution throughout the whole neuroblast layer (C). At P5 in the centre of the retina few Ki67+ cells were detected, whereas in Crb1Crb2 cKO retina many cells were still dividing especially in M-phase (D). Immunostaining of E17.5 retinal sections of control and Crb1Crb2 cKO was performed with Ki67 and BrdU antibodies after 30 min pulse (E) or 24 hours (F) of BrdU labelling and counterstained with DAPI for nuclear staining. Ki67 and BrdU positive cells localized through the entire thickness of Crb1Crb2 cKO. Flow cytometry profiles of control and Crb1Crb2 cKO retinal cells at E17.5 (G) based on Ki67 labelling and DNA content. The number of total proliferating cells using Ki67 immunostaining is increased by a factor two in Crb1Crb2 cKO retinas at P5 (H). Scale bar: 50 µm. ***P<0.001. (TIF) [file pgen.1003976.s006.tif]

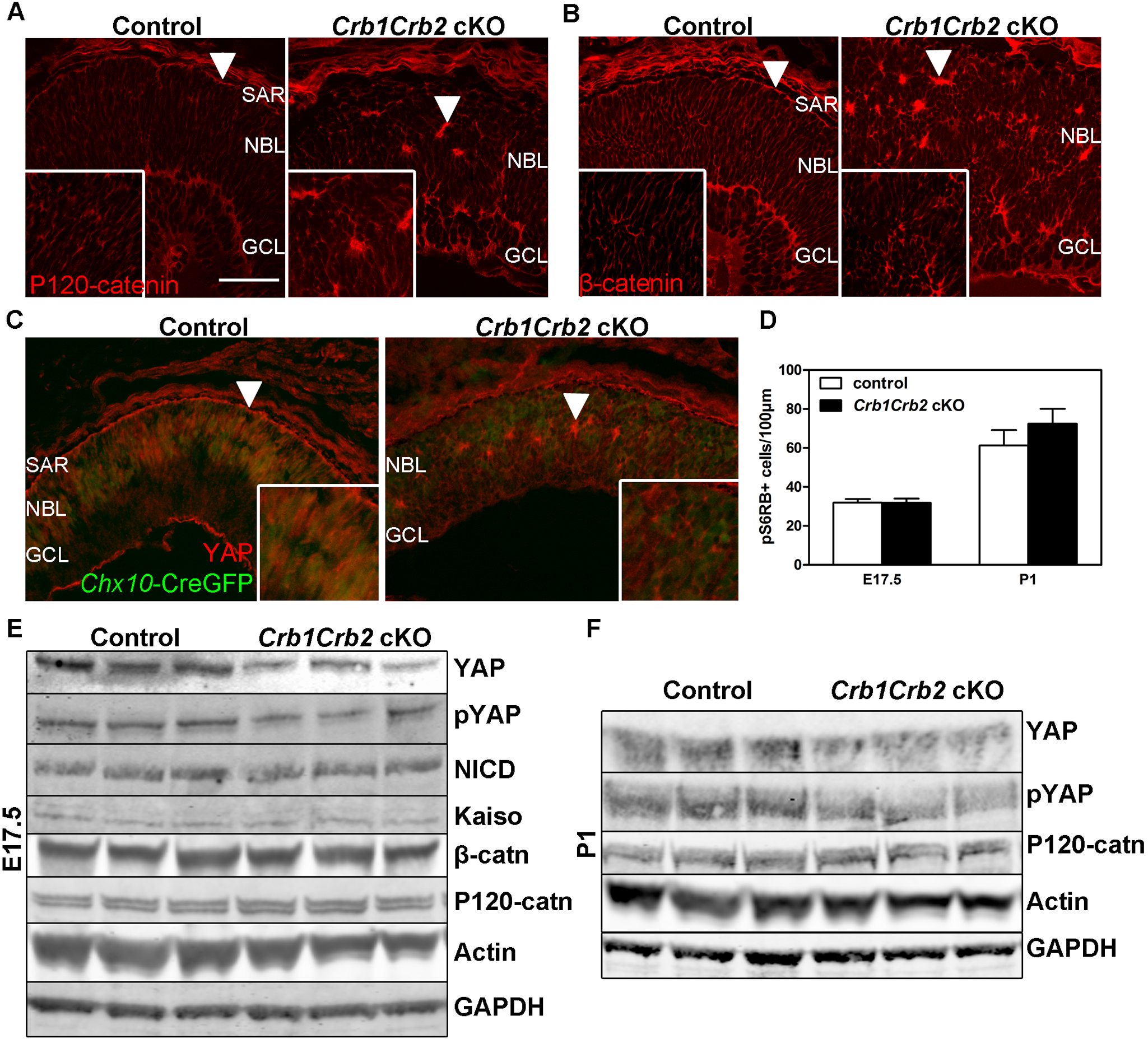

Supplement: Figure S7 — CRB1 and CRB2 act on proliferative signalling pathways. Representative pictures of P120-catenin (A), β-catenin (B) and YAP with Chx10Cre-GFP (C) of control and Crb1Crb2 cKO retinas at E17.5. P120- and β-catenins, which normally localized at the adherens junctions were found in ectopic rosette structures (white arrowheads). YAP protein normally localized at the subapical region adjacent to adherens junctions and in the nuclei of the retinal progenitors (colocalization with Chx10Cre-GFP) whereas YAP was found only in ectopic rosette structures (white arrowheads) similarly to the catenins and in the cytoplasm of the retinal progenitors in Crb1Crb2 cKO retinas. Quantification of the number of phospho-ribosomal protein positive cells (D) showed no difference between control and Crb1Crb2 cKO retinas at E17.5 and P1. Representative Western Blot of YAP, pYAP, Notch intracellular domain (NICD), Kaiso, β-catenin (β-catn), P120-catenin (P120-catn), actin and Glyceraldehyde 3-phosphate dehydrogenase (GAPDH) at E17.5 (E) and P1 (F). Scale bar: 50 µm. Values are presented as mean ± s.e.m. (TIF) [file pgen.1003976.s007.tif]
