# Supplementary material for: Targeted Ablation of Crb1 and Crb2 in Retinal Progenitor Cells Mimics Leber Congenital Amaurosis
Source: PLoS Genet. 2013 Dec 5;9(12):e1003976. doi: 10.1371/journal.pgen.1003976 (PMC3854796; doi:10.1371/journal.pgen.1003976)
Supplement: Table S1 — Antibody list and dilution used for immunohistochemistry. (DOC) [file pgen.1003976.s008.doc]

| | anti-goat-cy3 | donkey | 1/50 | Jackson Immunoresearch | | --- | --- | --- | --- | | anti-mouse-A488 | goat | 1/500 | Jackson Immunoresearch | | anti-mouse-cy3 | goat | 1/500 | Jackson Immunoresearch | | anti-rabbit-A488 | goat | 1/500 | Jackson Immunoresearch | | anti-rabbit-cy3 | goat | 1/500 | Jackson Immunoresearch | | anti-rat-cy3 | goat | 1/500 | Jackson Immunoresearch | | BrdU | rat | 1/40 | Abcam | | Brn3b | goat | 1/50 | Santa Cruz Biotechnology | | Calbindin (Calb) | rabbit | 1/400 | AnaSpec | | calretinin | rabbit | 1/500 | Chemicon | | cleaved caspase 3 (cCaspase3) | rabbit | 1/250 | Cell Signaling | | β-catenin | mouse | 1/100 | BD Biosciences | | P120-catenin | mouse | 1/100 | BD Biosciences | | Choline acetyltransferase (ChAT) | goat | 1/50 | Chemicon | | cone arrestin (CAR) | rabbit | 1/500 | Millipore | | GABA | rabbit | 1/300 | InStar | | Glutamine Synthetase (Gln Synth) | mouse | 1/200 | BD Biosciences | | Glycinergic Transporter 1 (GlyT1) | rabbit | 1/300 | Chemicon | | Ki67 | mouse | 1/50 | BD Biosciences | | MUPP1 | mouse | 1/200 | BD Biosciences | | Nectin1 | rat | 1/200 | MBL | | PALS1 | rabbit | 1/1000 | Proteintech | | PAR3 | rabbit | 1/100 | Upstate | | phospho-Histone H3 (pH3) | rabbit | 1/500 | Millipore | | phospho-S6RB (pS6RB) | rabbit | 1/250 | Cell Signaling | | phospho-YAP (pYAP) | rabbit | - | Cell Signaling | | PKCα | mouse | 1/200 | BD Transduction Laboratories | | PSD95 | rabbit | 1/200 | Cell Signaling | | Rhodopsin (RHO) | mouse | 1/250 | Millipore | | Sox9 | rabbit | 1/250 | Millipore | | YAP | rabbit | 1/200 | Cell Signaling | |
| --- | --- | --- | --- | --- | --- | --- | --- | --- | --- | --- | --- | --- | --- | --- | --- | --- | --- | --- | --- | --- | --- | --- | --- | --- | --- | --- | --- | --- | --- | --- | --- | --- | --- | --- | --- | --- | --- | --- | --- | --- | --- | --- | --- | --- | --- | --- | --- | --- | --- | --- | --- | --- | --- | --- | --- | --- | --- | --- | --- | --- | --- | --- | --- | --- | --- | --- | --- | --- | --- | --- | --- | --- | --- | --- | --- | --- | --- | --- | --- | --- | --- | --- | --- | --- | --- | --- | --- | --- | --- | --- | --- | --- | --- | --- | --- | --- | --- | --- | --- | --- | --- | --- | --- | --- | --- | --- | --- | --- | --- | --- | --- | --- | --- | --- | --- | --- | --- | --- | --- | --- | --- | --- | --- | --- |
