# Supplementary material for: Targeted Ablation of Crb1 and Crb2 in Retinal Progenitor Cells Mimics Leber Congenital Amaurosis
Source: PLoS Genet. 2013 Dec 5;9(12):e1003976. doi: 10.1371/journal.pgen.1003976 (PMC3854796; doi:10.1371/journal.pgen.1003976)
Supplement: Text S1 — Detailed Materials and Methods of In vivo analysis, immunohistochemical analysis and flow cytometry. (DOC) [file pgen.1003976.s009.doc]

**TEXT S1**

***In vivo* analysis.** Scanning laser ophthalmoscopy (SLO), spectral domain optical coherence tomography (SD-OCT) and electroretinography (ERG) measurements were performed at 1, 3, 6 and 12 month(s). The groups consisted of in 4 to 6 animals of each genotype. Electroretinograms were recorded binocularly as described previously [45]. Mice were anaesthetized using ketamine (66.7 mg/kg body weight) and xylazine (11.7 mg/kg body weight). The pupils were dilated and single-flash responses were obtained under scotopic (dark-adapted overnight) and photopic (light-adapted with a background illumination of 30 cd/m^2^ starting 10 minutes before recording) conditions. Single white-flash stimuli ranged from −4 to 1.5 log cd s/m^2^ under scotopic and from −2 to 1.5 log cd s/m^2^ under photopic conditions. Ten responses were averaged with inter-stimulus intervals of 5 s (for -4 to -0.5 log cd s/m^2^) or 17 s (for 0 to 1.5 log cd s/m^2^). Retinal morphology of the anesthetized animals was visualized via SLO imaging with a HRA 1 (Heidelberg Engineering, Heidelberg, Germany) according to previously described procedures [46]. Briefly, HRA 1 system feature two lasers (488/514 nm) in the short (visible) wavelength range and two (795/830 nm and 785/815 nm) in the long (infrared) wavelength range. For fundus visualization the 514 nm (RF, red-free) laser was used. The 488 and 795 nm lasers are used for fluorescein (FLA) and indocyanine green (ICG) angiography, respectively whereas autofluorescent images were obtained with the 488 nm laser (AF) with a barrier filter at 500 nm. In the same session, SD-OCT imaging was performed with a commercially available Spectralis HRA+OCT device from Heidelberg Engineering. This equipment features a broadband superluminescent diode at λ=870 nm as low coherent light source [47]. Each two-dimensional B-Scan recorded with the equipment set to 30° field of view, consists of 1,536 A-scans acquired at a speed of 40,000 scans per second. Optical depth resolution is approximately 7 μm with digital resolution reaching 3.5 μm. Imaging was performed using the proprietary software package Eye Explorer (version 3.2.1.0, Heidelberg Engineering) and for the work-up of the images we used CorelX3 (Corel Corporation, Ottawa, ON, Canada).

**Immunohistochemical analysis.** Eyes were enucleated and fixed during 20 minutes in 4% paraformaldehyde in PBS. Subsequently, the tissues were cryo-protected with 30% sucrose in PBS, embedded in Tissue-Tek O.C.T Compound (Sakura, Finetek) and used for cryosectioning. Cryosections (7 µm) were rehydrated in PBS and blocked for 1 hour using 10% goat or donkey serum, 0.4% Triton X-100 and 1% bovine serum albumin (BSA) in PBS. The antibodies used are listed in **Table S1**. The primary antibodies were diluted in 0.3% goat or donkey serum, 0.4% Triton X-100 and 1% BSA in PBS and incubated overnight at 4°C or 2 hours at room temperature in a moist chamber. Secondary antibodies were diluted in 1% BSA in PBS and incubated for 1 hour at room temperature in a moist chamber. Nuclei were counterstained and mounted in Vectashield hardset mounting medium containing DAPI (Vector Laboratories). Images were generated on a Leica epifluorescence and bright field microscope (DMRD) or a Leica SP5 confocal laser scanning microscope (CLSM) and analysed with Adobe Photoshop CS4.

**Flow cytometry.** These experiments were performed similarly to [29]. Retinas from at least 4 controls and *Crb1Crb2* cKO were isolated and mechanically dissociated with colagenase/DNAse I (370 U) at 37°C. Cells were fixed with 4% paraformaldehyde in PBS for 5 minutes followed by fixation in ethanol 70% one hour at 4°C. Cells were labelled with KI67 antibody diluted 1/50 in PBS-0.5% Tween-20-BSA 0.1% (PBS-TB) overnight at 4°C followed by goat anti-mouse-Alexa 488 antibody diluted 1/500 in PBS-TB. DNA content was labelled with PBS-TB containing 100 μg/ml RNase A 30 minutes at 37°C followed by 100 μg/ml propidium iodide 30 minutes. Cells analysis was performed using the flow cytometer BD LSR Fortessa. FACS data were analyzed using FlowJo software. Cells were gated for the single cell populations on the propidium iodide -W by propidium iodide -H on linear scale, followed by a gate for the DNA content on the propidium iodide-A by histogram. The cells were then plotted on their DNA content (propidium iodide-A; linear scale; X-axis) by KI67 labelling (KI67-A; Log scale; Y-axis). In the KI67 positive population, cells which have two times DNA content are in G2/M, cells which have only one time DNA content are in G1 and the cells in between are in S-phase. The KI67 negative population with only one DNA content are the cells that exit the cell cycle and are in G0 phase. Number of events is 10.000 at E17.5, 35.000 at P1 and 100.000 at P5 for each control and *Crb1Crb2* cKO.
